# Beyond integration: a coherence and transparency framework for mixed methods research in the era of generative AI

**DOI:** 10.3389/fpsyg.2026.1971387

**Published:** 2026-10-21

**Authors:** Rune Johan Krumsvik, Marius Ole Johansen

**Affiliations:** Department of Education, Faculty of Psychology, University of Bergen, Bergen, Norway

**Keywords:** coherence, ecological validity, generative artificial intelligence, integration, methodological pluralism, methodological transparency, mixed methods research, psychological science

## Abstract

Mixed methods research (MMR) has developed sophisticated approaches for combining qualitative and quantitative inquiry through design, integration, joint displays, and meta-inference. However, integration alone does not ensure methodological quality. A mixed methods study may demonstrate identifiable points of integration while retaining inconsistencies between its philosophical assumptions, theoretical framework, research questions, design, methods, analyses, and resulting meta-inferences. At the same time, the expanding use of generative artificial intelligence (GenAI) introduces new analytical intermediaries and potentially new points of integration, making methodological transparency, provenance, and human epistemic accountability increasingly consequential. In this conceptual analysis, we examine the relationships among methodological pluralism, integration, fit, alignment, coherence, ecological validity, and transparency and propose the Mixed Methods Coherence and Transparency Framework (MMCTF). The framework conceptualizes mixed methods quality across five interconnected domains: epistemic coherence, theoretical and design coherence, integrative coherence, inferential and ecological coherence, and methodological transparency. We distinguish coherence from both alignment and convergence, arguing that methodologically productive mixed methods inquiry may generate confirmation, expansion, or theoretically informative discordance. We further conceptualize GenAI not merely as an analytical tool but as a potential mediator of mixed methods integration whose influence must remain traceable and subject to human validation. The MMCTF is proposed as a conceptual architecture for supporting researchers, reviewers, and editors in designing and evaluating methodologically coherent and transparent mixed methods research, particularly as human and AI-supported analytical processes become increasingly intertwined in psychological research.

## 1 Introduction

Mixed methods research (MMR) has developed from debates concerning the compatibility of qualitative and quantitative traditions into a sophisticated methodological field concerned with the purposeful integration of different forms of evidence. Rather than treating qualitative and quantitative research as mutually exclusive methodological traditions, MMR seeks to combine their respective epistemic and analytical affordances in ways that generate insights that would be difficult to obtain through either approach alone (Johnson and Onwuegbuzie, 2004; Johnson et al., 2007; Greene, 2007; Teddlie and Tashakkori, 2009).

For analytical purposes, the development of contemporary MMR can be understood as involving three overlapping methodological emphases. An initial emphasis concerned the justification, classification, and architecture of mixed methods designs, including distinctions among convergent, explanatory sequential, exploratory sequential, embedded, and other designs. This work established a vocabulary through which researchers could articulate the purpose, priority, timing, and sequencing of qualitative and quantitative components.

A second emphasis shifted attention from the presence of multiple methods toward integration as the defining methodological feature of MMR. Fetters et al. (2013), for example, distinguished integration through connecting, building, merging, and embedding and demonstrated how integration can occur at design, methods, interpretation, and reporting levels. Subsequent work has further developed joint displays and other integration strategies that enable researchers to make the relationship between qualitative and quantitative evidence analytically visible (McCrudden et al., 2021). Schoonenboom (2022, 2023) has extended this perspective by directing attention beyond the integration of methods toward the successive integration of claims and the relationships between methods and inferences.

A third and increasingly prominent emphasis concerns not merely whether integration occurs, but the quality, integrity, and inferential consequences of integration. Fàbregues et al. (2024) provide a particularly important contribution through the Mixed Methods Integration Quality Framework (MMIQF). Derived from a methodological review of 135 publications, the MMIQF comprises 44 criteria across planning/data collection/analysis, interpretation, reporting, and joint displays and shifts attention from detecting integration toward evaluating how rigorously and transparently integration is implemented. Younas et al. (2025) extend this development further by conceptualizing different types of meta-inferences and emphasizing that the added value of MMR resides in the conclusions generated through the relationship between qualitative and quantitative inferences rather than in the simple coexistence of two datasets.

These developments mark an important maturation of the field. Integration is increasingly understood as both a process and a product, and its quality cannot be evaluated independently of the broader research architecture within which it occurs. The MMIQF explicitly recognizes that integration should be consistent with other components of an MMR study and that methodological and reporting quality need to be considered together (Fàbregues et al., 2024). At the same time, emerging work on meta-inferences demonstrates that integration may produce confirmation, complementarity, expansion, or discordance rather than simple convergence (Younas et al., 2025).

Nevertheless, an important conceptual question remains. What makes the complete mixed methods research architecture coherent? Integration quality primarily concerns the integrity of the processes through which qualitative and quantitative components are brought into relationship. Coherence, as conceptualized in the present article, concerns a broader set of relationships: whether the phenomenon under investigation, epistemological assumptions, theoretical framework, research questions, MMR purpose and design, qualitative and quantitative methodological traditions, integration mechanisms, and resulting meta-inferences form an explicit and defensible research architecture. Meta-inference refers here to the higher-order conclusion generated through the integration and interpretation of qualitative and quantitative evidence.

Accordingly, the proposed Mixed Methods Coherence and Transparency Framework (MMCTF) is not intended to replace the MMIQF or existing integration frameworks. Rather, it operates at a different conceptual level. Whereas the MMIQF provides detailed criteria for appraising the quality of integration, the MMCTF asks whether the methodological and epistemic components that integration connects are themselves coherently related and whether these relationships are sufficiently transparent to permit critical evaluation. In this sense, integration quality is conceptualized as a necessary component of, rather than a synonym for, overall methodological coherence.

The concept of coherence also requires differentiation from alignment and congruence. Alignment generally concerns the correspondence between particular methodological elements -for example, whether research questions align with methods or whether theoretical assumptions align with analytical procedures. Integration quality concerns the rigor with which qualitative and quantitative components are brought into relationship. Coherence is broader and relational: it concerns whether these multiple alignments and integrations collectively constitute a defensible research architecture. Thus, alignment can be understood as a local relationship between methodological components, integration as the purposeful interface between components, and coherence as a higher-order property of the research architecture as a whole.

Reporting transparency constitutes a related but distinct development. The original Good Reporting of a Mixed Methods Study (GRAMMS) guidance emphasized justification for mixing, description of design and individual methods, specification of where and how integration occurred, and articulation of the insights gained through mixing. An updated GRAMMS 2.0 is currently under development, using a methodological review and modified Delphi process to reflect subsequent advances in integration, fit, meta-inferences, joint displays, methodological quality, and reporting transparency (Munce et al., 2026). Importantly, GRAMMS 2.0 should at present be understood as an ongoing reporting-guideline development initiative rather than a finalized reporting standard.

## 2 Mixed methods coherence in psychological science

The need to move from integration toward coherence is particularly salient in psychological science. Psychology has historically been characterized by a strong preference for quantitative approaches, despite the capacity of qualitative inquiry to address dimensions of psychological phenomena that cannot readily be reduced to variables or population-level associations (Syed and Wilkinson Westberg, 2025). Mixed methods research therefore offers more than methodological diversification; it provides an architecture for bringing different forms of psychological knowledge into a purposeful relationship.

A particularly important tension concerns the relationship between nomothetic and idiographic knowledge. Psychological research frequently seeks generalizable patterns across individuals through standardized measurement, psychometric instruments, experimental designs, and statistical modelling. At the same time, psychological phenomena are experienced by particular persons within temporal, interpersonal, cultural, and situational contexts. Recent scholarship has consequently challenged a simple opposition between nomothetic and idiographic approaches and emphasized their potential complementarity (Kuper et al., 2025; Rauthmann et al., 2026). From an MMR perspective, this creates an important methodological question: how can population-level patterns and person- or context-sensitive evidence be brought into relationship without reducing one form of knowledge to the epistemic assumptions of the other?

This problem becomes particularly visible at the point of integration. For example, a psychometric measure may indicate a population-level association between two psychological constructs, whereas qualitative interviews may reveal substantial heterogeneity in how participants experience, interpret, or enact those constructs. Methodological coherence does not require the qualitative evidence to confirm the quantitative relationship, nor does it require lived experience to be transformed into variables merely to achieve convergence. Instead, the researcher must make explicit why the two forms of evidence are being related, what epistemic status is assigned to each, and how their relationship informs the resulting meta-inference.

This distinction is central to the MMCTF. Coherence should not be confused with epistemological homogenization. A coherent mixed methods study may deliberately preserve methodological and epistemic differences between its components. The criterion is not whether qualitative and quantitative evidence speak the same methodological language, but whether the relationship between them is theoretically justified, methodologically explicit, and inferentially productive. In psychological science, MMCTF therefore provides a framework for examining whether nomothetic patterns, idiographic experiences, theoretical assumptions, research questions, analytical procedures, and meta-inferences form a defensible research architecture.

Consider, for example, a mixed methods study of psychological treatment. Standardized symptom measures may indicate a statistically meaningful average improvement following an intervention, while qualitative interviews reveal that some participants experience little meaningful improvement, unexpected adverse experiences, or benefits in domains not represented by the psychometric instrument. These findings are not necessarily methodologically inconsistent. Rather, their relationship raises questions about the level of inference, construct representation, individual heterogeneity, and ecological meaning of the measured outcome. A coherent mixed methods interpretation must preserve these differences while explaining how they jointly inform the meta-inference.

The methodological relevance of integrating nomothetic and idiographic evidence is also evident in psychological research, where mixed methods studies have combined clinical and quantitative outcomes with clients’ perspectives in psychotherapy research (Bartholomew and Lockard, 2018), while more recent mental health research demonstrates how qualitative findings can inform quantitative analyses and how distinct methodological strands can be integrated into higher-order meta-inferences (Demkowicz et al., 2025).

Importantly, the presence of integration does not in itself demonstrate coherence across the wider research architecture. Conceptually, a study may contain identifiable points of integration while relationships among its epistemological assumptions, theoretical framing, research questions, methodological procedures, and resulting inferences remain insufficiently articulated. This concern is consistent with empirical methodological reviews showing substantial variation in how MMR rationale, design, integration, and methodological rigor are reported across psychological and related research fields (Bartholomew and Lockard, 2018; McCrudden et al., 2025; Demkowicz et al., 2025; Ketsman et al., 2025).

Recent evidence from educational psychology illustrates this distinction. In a systematic methodological review of 57 empirical MMR studies, McCrudden et al. (2025) found that researchers generally reported methodological rigor effectively, but also identified substantial variation across reporting criteria. For example, 35.1% of the studies provided no identifiable rationale for using mixed methods, while 31.6% neither discussed mixed methods nor referenced the MMR literature. Such findings should not be interpreted as evidence that these studies were necessarily methodologically incoherent; rather, they demonstrate a transparency problem: when key methodological relationships remain unreported, readers cannot fully evaluate whether coherence has been achieved.

Comparable reporting challenges have been identified beyond psychology; for example, Ketsman et al. (2025) found that 64% of identified MMR studies in leading educational technology journals did not self-identify as mixed methods, while 68% of those that did self-identify failed to specify their core MMR design.

These findings reinforce a central premise of the MMCTF: methodological coherence and methodological transparency are analytically distinct but mutually dependent quality dimensions. A study may be coherent without reporting that coherence adequately, but insufficient transparency makes coherence difficult—or impossible—for readers, reviewers, and editors to evaluate.

Importantly, coherence should not be confused with convergence. A coherent mixed methods study does not require qualitative and quantitative findings to confirm one another. Integration may instead generate confirmation, complementarity, expansion, or discordance, each of which may contribute to theoretically meaningful meta-inferences when appropriately investigated and justified (Younas et al., 2025). Discordance can be particularly informative because apparent contradictions may reveal differences in constructs, levels of analysis, temporal processes, contexts, or participant experiences that would remain obscured within a single methodological strand. The criterion for coherence is therefore not agreement between findings, but whether their relationship is explicitly examined and incorporated into a defensible meta-inference.

This position is consistent with arguments that mixed methods inquiry should not seek prematurely to resolve complexity, contradiction, or dissonance merely to produce a unified account; differences between forms of evidence may themselves constitute analytically valuable findings (Uprichard and Dawney, 2019).

For example, returning to the psychological intervention example introduced above, a psychological intervention may produce a statistically significant mean reduction in symptom scores while qualitative interviews reveal that some participants experience little improvement, adverse effects, or meaningful benefits in domains not captured by the standardized measure. Such findings are discordant only in a limited sense: they may reflect different levels of analysis or different representations of the psychological phenomenon. Within the MMCTF, methodological coherence requires neither dataset to be privileged simply to obtain convergence; rather, the discrepancy should become an object of integration and contribute to the meta-inference.

Against this background, this Conceptual Analysis has four objectives: (1) to differentiate methodological coherence from integration, integration quality, alignment, and convergence in MMR; (2) to develop the MMCTF as a higher-order conceptual architecture encompassing epistemic, theoretical and design, integrative, inferential and ecological, and transparency dimensions; (3) to examine how this framework addresses distinctive methodological and epistemic tensions in psychological science; and (4) to analyze how generative and predictive AI may introduce new analytical interfaces and points of integration while increasing requirements for transparency, provenance, human validation, and epistemic accountability.

The article proceeds by first examining methodological pluralism and the development from design-oriented MMR toward integration quality and meta-inference. It then develops the concept of coherence and operationalizes the five domains of the MMCTF, including their vertical and horizontal relationships. Subsequently, the framework is considered in relation to psychological science and AI-mediated research before its implications for researchers, reviewers, editors, and research-methods education are discussed.

## 3 Methodological foundations and conceptual development of the MMCTF

### 3.1 Methodological pluralism beyond the paradigm wars

The development of mixed methods research is partly rooted in the movement away from strict methodological purism and the incompatibility thesis that characterized parts of the historical quantitative–qualitative “paradigm wars.” From a pluralistic perspective, the principal question is not whether qualitative or quantitative research represents the intrinsically superior methodological paradigm, but which forms of inquiry are appropriate for the phenomenon and questions under investigation (Johnson and Onwuegbuzie, 2004; Greene, 2007; Johnson, 2017).

Methodological pluralism does not imply methodological relativism or the assumption that all methods are equally suitable for all purposes. Neither does it mean that combining multiple approaches is inherently superior to conducting a rigorous monomethod study. Instead, methodological pluralism recognizes that research traditions provide different and sometimes complementary forms of epistemic access to complex phenomena.

This distinction is crucial. Without it, methodological pluralism can deteriorate into methodological opportunism: researchers may add methods because data or analytical techniques are available rather than because the research problem requires them.

Krumsvik (2020), discussing methodological pluralism in educational technology research, emphasized this tension by describing MMR as potentially valuable but methodologically demanding. The problem is not solved by merely adding qualitative interviews to a quantitative survey, or vice versa. The methodological rationale for the relationship between components needs to be explicit.

A useful distinction is therefore between methodological multiplicity and methodological integration.

Multiple methods may increase the amount of evidence. Mixed methods research should increase the *relational value* of evidence.

### 3.2 The research question and the purpose of mixing as methodological anchor

A central principle across the MMR literature is that the research problem and research questions should drive methodological decisions. Schoonenboom and Johnson (2017) emphasize that the purpose of mixing must be determined in interaction with the research questions, and Greene et al. (1989) provide an enduring classification of five major purposes for mixing: triangulation, complementarity, development, initiation, and expansion.

These purposes differ substantially. Triangulation seeks convergence or corroboration. Complementarity seeks elaboration and clarification. Development uses findings from one method to inform another. Initiation deliberately searches for contradiction, paradox, or alternative perspectives. Expansion extends the range of inquiry by assigning different components to different dimensions of a phenomenon. Consequently, mixed methods should not be reduced to a universal aspiration toward agreement between data sources.

Hesse-Biber et al. (2015) further demonstrate why theoretical and epistemological drive matters. A qualitatively driven mixed methods design is not equivalent to an equal-status design. Rather, the qualitative epistemology and methodology provide the central organizing logic, with quantitative methods fulfilling an auxiliary function. Qualitative and quantitative components can therefore legitimately have different weights and functions within the same research architecture.

When studying suicidal behavior and suicide prevention (e.g., Krumsvik et al., 2026), a primarily qualitative study of lived experiences of depression may use quantitative prevalence or symptom measures to contextualize participants’ accounts. Conversely, an intervention study may be quantitatively driven but use qualitative inquiry to understand why particular participants respond or fail to respond to treatment. The key quality criterion is not symmetry. It is methodological justification.

### 3.3 Conceptual development of the MMCTF

The MMCTF was developed through a structured conceptual synthesis and framework-development process (as well as 10 years of MMR-teaching on doctoral level), rather than through an empirical validation study or systematic review. This approach is consistent with the purpose of conceptual research, in which existing constructs and literature streams are analyzed, differentiated, and recombined to generate new conceptual relationships and higher-order explanatory structures (Jabareen, 2009; Jaakkola, 2020). The framework should therefore be understood as a theoretically derived and proposition-generating conceptual architecture whose empirical utility remains open to subsequent evaluation.

The development proceeded through four iterative analytical phases. First, we identified established constructs central to contemporary MMR scholarship, including methodological pluralism, theoretical drive, design, integration, points of integration, fit, joint displays, legitimation, meta-inference, ecological validity, and methodological transparency. The purpose was not to conduct an exhaustive systematic review, but to map the principal conceptual domains necessary for examining relationships across the complete mixed methods research architecture.

Second, these constructs were subjected to conceptual differentiation and relational analysis. Particular attention was given to constructs that are related but not synonymous, including alignment, integration, integration quality, convergence, coherence, and transparency. This phase revealed an analytical distinction between relationships among particular methodological components, interfaces between qualitative and quantitative strands, and the higher-order relationship among the components of the research architecture as a whole.

Third, the identified relationships were synthesized into five higher-order domains: epistemic coherence; theoretical and design coherence; integrative coherence; inferential and ecological coherence; and methodological transparency. The domains were iteratively examined against established MMR frameworks and methodological scholarship to determine whether they represented conceptually distinguishable dimensions while remaining relationally connected. Existing frameworks addressing integration quality, points of integration, meta-inference, legitimation, and reporting were treated as complementary benchmarks rather than as frameworks to be replaced.

Fourth, the emerging framework was examined through conceptual stress-testing against methodological situations in which integration does not automatically imply coherence. These included discordant qualitative and quantitative findings, differences in epistemological assumptions across methodological strands, tensions between population-level and person- or context-sensitive psychological evidence, multiple points of integration, and the introduction of AI-mediated analytical processes. The purpose of this phase was to assess whether the framework could preserve theoretically productive methodological differences while identifying relationships that require explicit justification and transparency.

The resulting MMCTF therefore represents a higher-order relational framework rather than a new MMR design taxonomy or reporting guideline. Its purpose is to make visible the relationships through which methodological coherence is established and evaluated across an MMR study. Following Jaakkola’s (2020) terminology, the conceptual design combines elements of theory synthesis and model development. At this stage, the MMCTF should therefore be considered conceptually developed rather than empirically validated; future research should examine its usability, discriminant value, and inter-rater applicability across different MMR designs and disciplinary contexts. The four phases of conceptual development are summarized in Table 1.

**Table 1:** Conceptual development of the MMCTF.

Phase	Conceptual procedure	Purpose	MMCTF outcome
1. Concept identification	Map established MMR constructs	Identify relevant conceptual building blocks	Initial conceptual domain
2. Differentiation	Compare alignment, integration, fit, convergence, coherence, transparency	Establish conceptual boundaries	Definition of coherence
3. Relational synthesis	Organize concepts into higher-order relationships	Construct framework architecture	Five MMCTF domains
4. Conceptual stress-testing	Examine discordance, epistemic tension, multiple integration points, psychology and AI-mediated analysis	Test conceptual robustness and boundary conditions	Refined MMCTF and propositions

### 3.4 Operationalizing the MMCTF: from methodological tension to meta-inference

The five MMCTF domains are not intended merely as retrospective quality criteria. They can also function prospectively as a decision sequence when methodological tensions emerge during an MMR study. Such tensions should not automatically be treated as methodological failures. Rather, they may signal differences in epistemological assumptions, construct representation, levels of analysis, contextual conditions, or participant experiences that require further integration. This is particularly important when qualitative and quantitative findings are discordant, because suppressing discordance may eliminate precisely the complexity that MMR is intended to reveal.

We propose a five-step MMCTF decision sequence:

1. Epistemic diagnosis: researchers identify what kind of knowledge each strand is producing and whether the apparent discrepancy reflects different epistemic purposes or levels of inference.
2. Theoretical and design diagnosis: researchers examine whether both strands address the same theoretical construct, population, temporal frame, and research question, and whether the observed relationship is consistent with the intended MMR design.
3. Integrative diagnosis: the point and mechanism of integration are made explicit, and researchers determine whether connecting, building, merging, embedding, data transformation, joint display, or another strategy is most appropriate for interrogating the relationship between the findings.
4. Inferential and ecological diagnosis: researchers examine whether the discrepancy identifies heterogeneity, contextual boundary conditions, construct limitations, or differences between population-level patterns and lived experience.
5. Transparency and meta-inference: the analytical decisions, unresolved tensions, and evidential contribution of each strand are documented, and the resulting meta-inference explicitly communicates what became knowable through integration that could not have been inferred from either strand independently.

This sequence means that MMCTF does not provide an algorithm for forcing methodological differences into agreement. Rather, it provides a structured process for determining whether a tension represents methodological incoherence that requires correction or substantively meaningful discordance that should be preserved and explained. The latter may constitute an important source of mixed methods added value.

Returning to the psychological intervention example introduced above - consider a mixed methods evaluation of a psychological intervention in which standardized symptom measures indicate a statistically significant average improvement, whereas qualitative interviews reveal that a subgroup of participants experiences little meaningful benefit and describes important outcomes that are not represented by the psychometric instrument. Treating this discrepancy merely as a failure of convergence would provide little methodological insight.

Applying the MMCTF, the epistemic-coherence step first asks whether the two strands are making claims at the same level: the quantitative component may estimate average change in predefined constructs, whereas the qualitative component may capture person-specific meanings and experiences. The theoretical/design step examines whether the psychometric construct adequately represents the phenomenon described by participants and whether sampling or timing differences may account for part of the discrepancy. At the integrative step, the researcher might construct a joint display linking symptom trajectories with qualitative accounts, purposefully examine cases that diverge from the aggregate pattern, or use qualitative findings to guide subsequent subgroup analyses. The inferential/ecological step then considers whether the qualitative evidence identifies boundary conditions, heterogeneity, contextual influences, or outcomes omitted from the standardized measure. Finally, the transparency step requires the researcher to document these analytical decisions and preserve unresolved discordance in the final interpretation.

The resulting meta-inference might therefore be that the intervention is associated with average symptom improvement while its perceived benefits vary across participants and include dimensions not fully represented by the standardized outcome measure. This meta-inference is neither exclusively quantitative nor qualitative and does not require artificial convergence. It is generated through their relationship.

MMIQF asks whether integration has been implemented with sufficient quality; MMCTF additionally asks what researchers should do when the relationship among the components being integrated exposes a potential coherence problem (illustrated in Table 2). Table 2 operationalizes the MMCTF as a diagnostic sequence when methodological friction emerges.

**Table 2:** The MMCTF-steps.

MMCTF step	Diagnostic question	Possible researcher action	Example
1. Epistemic diagnosis	Are the strands making the same kind and level of knowledge claim?	Identify epistemic purpose and level of inference	Population mean vs. lived experience
2. Theoretical/design diagnosis	Do both strands operationalize the phenomenon compatibly?	Re-examine constructs, sampling, timing and design	Symptom scale may omit meaningful outcomes
3. Integrative diagnosis	Where and how should the findings interact?	Merge, connect, build, embed, transform or use joint display	Link symptom trajectories to interview accounts
4. Inferential/ecological diagnosis	What does the relationship reveal?	Examine heterogeneity, context and boundary conditions	Identify subgroup or contextual differences
5. Transparency/meta-inference	What can be concluded from the integrated evidence?	Document decisions, preserve unresolved discordance and formulate meta-inference	Average improvement with heterogeneous experiences

The MMCTF therefore distinguishes between two broad outcomes of methodological friction: (a) correctable incoherence, where the discrepancy results from an unjustified mismatch among research questions, theoretical assumptions, design, methods, or analytical procedures; and (b) productive discordance, where appropriately conducted methodological strands generate different but defensible insights into the phenomenon. The former calls for methodological correction; the latter calls for integration, explanation, and preservation in the meta-inference.

### 3.5 Qualitative traditions and coherence at the point of integration

The designation “qualitative component” should not be interpreted as referring to a methodologically homogeneous category. Qualitative traditions differ in their ontological and epistemological assumptions, purposes, relationships between researcher and participant, approaches to sampling and analysis, and the kinds of knowledge claims they seek to generate. Consequently, the methodological implications of integrating qualitative and quantitative evidence depend not only on the type of MMR design but also on the qualitative tradition embedded within that design.

Phenomenologically oriented research, for example, prioritizes the meaning and structure of lived experience and therefore raises particular questions concerning the relationship between first-person experience and standardized or population-level measurement. Phenomenological mixed methods research should not reduce subjective experience to quantitative variables merely to facilitate integration; rather, the methodological challenge is to preserve the epistemic contribution of lived experience while establishing a theoretically defensible relationship with quantitative evidence (Mayoh and Onwuegbuzie, 2015; Martiny et al., 2021). From an MMCTF perspective, coherence therefore requires that integration remain consistent with the phenomenological commitments that justify the qualitative component in the first place.

Grounded theory presents a different integration logic because its primary purpose is theory generation rather than the description of lived experience. Integration may occur iteratively through theoretical sampling, constant comparison, development of categories, and subsequent elaboration or examination of an emerging theory using quantitative evidence. However, grounded theory itself encompasses different methodological traditions (Ratnapalan, 2019; Guetterman et al., 2019). Classical or Glaserian grounded theory places particular emphasis on emergence and minimizes premature theoretical imposition, whereas constructivist grounded theory emphasizes the co-construction of meaning and the interpretive role of the researcher. Clarke’s situational analysis further expands the analytical frame toward discourses, positions, social worlds, materialities, power relations, and complexity. These differences have consequences for what constitutes a coherent point of integration: an integration procedure compatible with one grounded theory tradition cannot automatically be assumed to be epistemically compatible with another.

These distinctions illustrate what we term tradition-sensitive coherence: the requirement that integration preserve the defining epistemological and methodological commitments of the qualitative tradition while making its relationship with the quantitative component explicit and defensible. Table 3 illustrates this principle across selected qualitative traditions. The examples are not intended as an exhaustive taxonomy but as demonstrations of how the meaning of coherence may vary according to the epistemic purpose and methodological commitments of the qualitative approach.

**Table 3:** Illustrations of qualitative methodological traditions and tradition-sensitive coherence.

Qualitative tradition	Primary epistemic contribution	Key integration challenge	MMCTF coherence question
Phenomenology	Meaning and structure of lived experience	Avoid reducing first-person experience and contextual meaning to predefined variables or decontextualized categories	Does integration preserve the epistemic integrity and contextual meaning of lived experience?
Classical/Glaserian grounded theory	Emergent theory generation grounded in patterns identified through iterative engagement with data	Avoid imposing predetermined theoretical categories or allowing integration to disrupt emergence, theoretical sampling, and constant comparison	Does integration support rather than prematurely constrain the emergence and development of grounded theory?
Constructivist grounded theory	Interpretive and co-constructed development of categories and theory	Avoid treating categories and theoretical interpretations as researcher-independent representations of an objective reality	Does integration preserve reflexivity, researcher positionality, and the co-constructed character of interpretation and theory development?
Situational analysis	Mapping relations, positions, discourses, social worlds, and complexity within situations	Avoid reducing relational complexity, multiple positions, silences, and differences to common variables or simplified categories	Does integration preserve relational complexity, multiple positions, and theoretically consequential differences within the situation?

The same principle applies to more complex and structured multimethod configurations. The MMCTF is not restricted to core convergent or sequential designs and can, in principle, be applied to designs incorporating iterative expert-elicitation procedures, mixed Delphi approaches, structural modelling, or multi-criteria decision procedures. In such configurations, the relevant question is not whether the procedure belongs to a predefined MMCTF taxonomy, but whether its epistemic purpose, sequencing, dependency, transformation of evidence, point(s) of integration, and contribution to the final meta-inference are coherent with the overall research architecture.

MMCTF is therefore design-flexible but coherence-constrained: methodological diversity is permitted, and often desirable, provided that the relationships among the components remain epistemically defensible, methodologically explicit, and inferentially productive.

## 4 From integration quality to methodological coherence

### 4.1 Integration is necessary but insufficient

Integration is a defining feature of MMR, but its presence does not by itself establish the methodological quality of the complete study. A study may contain clearly identifiable points of integration while important relationships elsewhere in the research architecture remain insufficiently justified. For example, qualitative themes and psychometric scores may be systematically combined in a joint display, demonstrating that integration has occurred, while the study nevertheless contains tensions among its research problem, theoretical assumptions, sampling strategy, analytical procedures, or resulting meta-inferences.

The converse is also possible. Qualitative and quantitative components may each be methodologically rigorous while their relationship remains conceptually weak or inferentially underdeveloped. It is therefore useful to distinguish among three related but analytically different dimensions of mixed methods quality:

*Component quality* concerns whether the qualitative and quantitative components are methodologically defensible in their own terms. *Integration quality* concerns whether and how these components are purposefully brought into relationship. *Overall coherence* concerns whether the complete research architecture constitutes an explicit and defensible whole.

The third cannot be reduced to the first two. Recent developments in MMR have substantially strengthened the conceptualization and appraisal of integration. The MMIQF, for example, provides criteria for evaluating integration quality across the research process (Fàbregues et al., 2024), while Bazeley (2024) conceptualizes integration as dynamic interdependence among methodological components. These developments make it possible to ask a further question: even when integration itself is methodologically strong, how coherent is the broader research architecture within which that integration occurs?

The MMCTF addresses this higher-order question. It does not replace integration-quality frameworks but extends the analytical lens beyond the integration process itself. We therefore propose the following foundational proposition:

*Integration is necessary but insufficient for methodological coherence.*

### 4.2 From alignment and fit to mixed methods coherence

We define mixed methods coherence as *the extent to which the philosophical, theoretical, methodological, analytical, integrative, and inferential components of a mixed methods study form an explicit and defensible relationship with one another and with the phenomenon and research questions being investigated.*

Coherence should be distinguished from related concepts such as alignment, integration, and fit. *Alignment* refers primarily to the correspondence between particular components of a study - for example, whether research questions are aligned with methods or whether analytical procedures are congruent with the underlying methodological assumptions. *Integration* concerns the purposeful interfaces through which qualitative and quantitative components are brought into relationship, including connecting, building, merging, embedding, transformation, and integration at the level of interpretation (Fetters et al., 2013; Schoonenboom, 2022, 2023). *Fit*, as used by Fetters et al. (2013), concerns principally the relationship between qualitative and quantitative findings, including confirmation, expansion, and discordance.

Coherence is broader than each of these concepts. It concerns whether the relationships and interfaces across the complete research architecture collectively form a defensible whole. This includes relationships among the research phenomenon, epistemological assumptions, theoretical framework, research questions, MMR purpose and design, sampling and methods, qualitative and quantitative analyses, points of integration, and resulting meta-inferences.

Accordingly, alignment may be understood as a relationship between particular components, integration as an interface between methodological strands, and coherence as a higher-order property of the research architecture constituted by these relationships and interfaces.

This broader conceptualization also distinguishes the MMCTF from approaches that locate coherence primarily within integration itself. Bazeley (2024), for example, conceptualizes coherence as a constitutive dimension of integration. The MMCTF extends the analytical scope by treating coherence as a higher-order property of the complete research architecture and by differentiating vertical, horizontal, and inferential coherence while positioning transparency as the condition through which these relationships can be critically evaluated.

Importantly, coherence does not require methodological homogeneity. Qualitative and quantitative inquiry may rest on different assumptions concerning evidence, meaning, context, explanation, and generalization. Methodological pluralism may deliberately preserve these differences and bring them into productive relationship (Greene, 2007; Johnson, 2017). Qualitatively driven MMR, for example, may retain the epistemological centrality of qualitative inquiry while incorporating quantitative evidence for a particular and explicitly bounded purpose (Hesse-Biber et al., 2015).

The relevant requirement is therefore not epistemological sameness but epistemic defensibility:

*Coherence does not require eliminating epistemological tensions; it requires recognizing, justifying, and methodologically managing them.*

This distinction is particularly consequential in psychological science, where population-level or psychometric evidence may be brought into relationship with idiographic and interpretive evidence concerning lived experience, meaning, context, and individual variation.

### 4.3 Coherence should not be confused with convergence

A further distinction concerns the relationship between coherence and convergence. An enduring intuition in MMR is that agreement between qualitative and quantitative findings represents a stronger outcome than disagreement. Convergence may indeed strengthen confidence in a finding when different forms of evidence independently support a similar conclusion. However, corroboration is only one possible purpose and outcome of mixed methods integration.

Mixed methods inquiry may also produce complementarity, expansion, initiation, or discordance (Greene et al., 1989; Fetters et al., 2013). More recent work on meta-inference similarly distinguishes confirmed, complementary, expanded, and discordant relationships between findings (Younas et al., 2025). These relationships should not be arranged as a simple hierarchy in which convergence is methodologically superior to difference.

Discordance may be especially informative because apparent contradictions can reveal differences in constructs, measurement, sampling, temporal processes, levels of analysis, contexts, subgroups, or participant experiences that would remain obscured within either methodological strand alone. The methodological response to discordance is therefore not automatically to determine which dataset is “correct” or to force the findings into a unified account. Rather, the discrepancy itself may become an object of integration.

This leads to a second foundational proposition:

*Coherence should not be confused with convergence.*

The criterion for coherence is not agreement between findings, but whether their relationship is explicitly examined, methodologically justified, and incorporated into a defensible meta-inference. A coherent MMR study may therefore produce findings that confirm one another, extend one another, or remain meaningfully discordant.

### 4.4 From methodological friction to correctable incoherence or productive discordance

The distinction between coherence and convergence creates an important diagnostic question. When different components of a mixed methods study do not fit comfortably together, does this indicate a methodological problem that should be corrected, or a substantive difference that should be preserved and explained?

The MMCTF conceptualizes such situations initially as *methodological friction*: a point at which assumptions, methods, findings, interpretations, or inferential claims appear to be in tension. Methodological friction is not itself evidence of either poor quality or valuable discordance. Its methodological significance must be diagnosed within the wider research architecture.

The framework therefore distinguishes between two broad outcomes. *Correctable incoherence* occurs when the tension reflects an unjustified mismatch - for example, between the research question and design, theoretical assumptions and operationalization, sampling and intended inference, qualitative tradition and analytical procedure, or integrated evidence and the claims subsequently made. Such friction calls for methodological reconsideration, redesign, reanalysis, qualification, or correction.

*Productive discordance*, by contrast, occurs when appropriately conducted methodological components generate different but defensible insights into the same phenomenon. Here, the difference is not a methodological defect to be removed but potentially a source of additional knowledge. The appropriate response is to investigate the discrepancy, consider alternative explanations, return where necessary to the underlying evidence, and incorporate the resulting understanding into the meta-inference.

This distinction is particularly important in psychological research. Population-level patterns or average intervention effects may coexist with substantial heterogeneity in individual experiences. Quantitative evidence may indicate an average reduction in psychological symptoms, for example, while qualitative accounts reveal that particular participants experienced limited benefit, unintended effects, or outcomes not represented by the primary measure. Such findings are not necessarily incoherent. They may indicate contextual or subgroup variation that qualifies the scope of the quantitative conclusion and produces a more defensible integrated interpretation.

The resulting logic can be summarized as:

*Methodological friction → MMCTF diagnosis → correctable incoherence OR productive discordance → methodological response → meta-inference.*

The aim of coherence is therefore not to eliminate epistemic or empirical tension. It is to determine what that tension signifies within the research architecture and to make it methodologically explicit and inferentially productive.

In this sense, coherence can be understood as a higher-order property of the research architecture that emerges from the relationships among its components rather than as a property located within any single component or point of integration.

## 5 The mixed methods coherence and transparency framework

We propose the *Mixed Methods Coherence and Transparency Framework (MMCTF)* as a higher-order conceptual framework for designing, reporting, and evaluating mixed methods research.

The framework comprises five interconnected domains:

1. Epistemic coherence
2. Theoretical and design coherence
3. Integrative coherence
4. Inferential and ecological coherence
5. Methodological transparency

These domains do not represent sequential stages. They are recursively connected and may require reconsideration throughout an interactive or emergent study.

### 5.1 Domain 1: epistemic coherence

Epistemic coherence concerns the relationship between the research phenomenon, ontological assumptions, epistemology, axiology, paradigm positioning, and the knowledge claims sought.

Relevant questions include:

- What kind of phenomenon is being investigated?
- Which dimensions of the phenomenon are assumed to be measurable, interpretable, contextual, relational, or constructed?
- What kinds of knowledge are required to address the research question?
- Why are both qualitative and quantitative perspectives needed?
- What philosophical or metaparadigmatic stance structures the relationship between these forms of evidence?
- Are differences between epistemological assumptions acknowledged?

MMR does not require an extensive philosophy-of-science treatise in every manuscript. The extent of explicit philosophical positioning should depend on the design and the epistemological tensions it entails.

Nevertheless, philosophical assumptions cannot simply disappear because a study adopts a pragmatic label.

Pragmatism itself does not mean that “anything goes.” Rather, methodological choices should remain related to inquiry purposes and consequences.

Epistemic coherence is therefore present when the kinds of knowledge claimed in the conclusion are defensible given the assumptions underlying how that knowledge was generated.

### 5.2 Domain 2: theoretical and design coherence

The second domain concerns alignment among:

*research problem ↔ theory ↔ research questions ↔ purpose of mixing ↔ theoretical drive ↔ MMR design.*

Theory should perform an identifiable function. In deductively driven research, theory may generate hypotheses or relationships for testing. In inductive research, theory may develop through engagement with empirical material. Abductive MMR may involve recursive movement between empirical findings and theoretical interpretation.

Theoretical eclecticism can be productive, particularly when complex psychological phenomena require multiple explanatory lenses, but it creates additional demands for justification. Combining theories merely because each explains one variable or dataset can produce conceptual fragmentation.

Design coherence asks whether the MMR architecture follows from the purposes of inquiry.

- Why explanatory sequential rather than convergent?
- Why qualitatively driven rather than equal status?
- Why collect the components concurrently if one will later inform interpretation of the other?
- Why is an emergent element necessary?
- Does the priority assigned to QUAL or QUAN correspond to the theoretical drive?

Complexity itself is neither a strength nor a weakness. A high-complexity design may be highly coherent, while a basic two-phase design may be incoherent.

### 5.3 Domain 3: integrative coherence

The third domain addresses the defining methodological relationship between QUAL and QUAN.

Key questions include:

- Why is integration needed?
- Where does it occur?
- When does it occur?
- How does it occur?
- What does integration add?

Identifying a point of integration is therefore necessary but not sufficient.

For *building,* researchers should specify which findings informed the subsequent instrument, categories, interview protocol, or hypotheses.

For *connecting,* they should explain how one dataset informed sampling for another.

For *merging,* the conceptual and analytical basis on which datasets are compared should be explicit.

For *embedding,* the multiple relationships between data collection and analysis should be traceable.

For *joint displays,* the display should produce integrative insight rather than merely place two results side by side.

The final question is crucial:

*What can be inferred from the integrated evidence that could not reasonably have been inferred from the separate strands alone?*

Without such added value, the methodological case for MMR remains incomplete.

### 5.4 Domain 4: inferential and ecological coherence

Inferential coherence concerns whether the conclusions drawn from the integrated evidence are warranted by the qualitative and quantitative components and by the relationship established between them. Ecological coherence extends this requirement by asking whether the resulting meta-inference remains plausible and appropriately bounded when situated within the contexts, populations, settings, and conditions in which the psychological phenomenon occurs.

Ecological coherence should not be understood simply as ecological validity in the conventional sense of whether findings generalize to real-world settings. Within the MMCTF, it concerns the relationship between contextual knowledge and the boundaries of inference. Qualitative evidence may identify contextual conditions, mechanisms, participant characteristics, temporal processes, or meanings that qualify an apparently general quantitative relationship. Conversely, quantitative evidence may indicate whether patterns identified qualitatively extend beyond particular cases or contexts. Ecological coherence is achieved when these different forms of evidence are integrated to specify not only whether an effect, association, or experience occurs, but also for whom, under what conditions, in which contexts, and with what limitations the resulting meta-inference is defensible.

Importantly, contextual evidence should not remain an interpretive appendix to the quantitative findings. When theoretically and methodologically justified, qualitative findings may generate propositions concerning moderators, subgroup differences, contextual boundary conditions, mechanisms, or alternative construct specifications that can subsequently inform quantitative analysis. This may involve examining interaction effects, stratified or subgroup analyses, longitudinal variation, alternative model specifications, or theoretically justified sensitivity analyses. The direction may also be reversed: unexpected quantitative heterogeneity may guide purposeful qualitative follow-up to identify contextual mechanisms or meanings that explain variation. Ecological coherence is therefore potentially bidirectional and iterative rather than a final-stage contextual commentary.

Consider again a psychological intervention that produces a statistically significant average reduction in symptom scores. Qualitative interviews may indicate that perceived benefit differs according to social support, treatment setting, previous treatment experience, or participants’ ability to incorporate the intervention into everyday life. Within the MMCTF, these findings should not automatically be treated merely as contextual observations. If theoretically warranted and supported by the study design and data, they may generate hypotheses concerning effect modification or boundary conditions that can be examined quantitatively. For example, social support may be examined as a moderator of treatment response, or treatment trajectories may be compared across theoretically meaningful subgroups.

If such analyses indicate that the intervention is particularly beneficial under specific contextual conditions, the resulting meta-inference becomes more ecologically coherent: rather than concluding simply that “the intervention works,” the integrated evidence supports a bounded claim specifying for whom, under which conditions, and in what contexts benefit is most plausible. If the available quantitative data do not permit such analyses, the qualitative evidence may instead define explicit boundary conditions for interpretation and hypotheses for subsequent research.

The MMCTF does not imply that every qualitative observation should be converted into a quantitative moderator or tested *post hoc*. Such analyses require theoretical justification, adequate measurement, sufficient statistical power, and transparent distinction between confirmatory and exploratory analyses. Where these conditions are not met, contextual qualitative evidence can still strengthen ecological coherence by bounding the scope of the meta-inference rather than by generating additional statistical tests.

Statistical generalizability asks how broadly a quantitative pattern may extend; ecological coherence asks whether the scope of the integrated meta-inference is consistent with what both the quantitative patterns and contextual evidence permit us to claim.

Inferential coherence concerns the relationship between evidence and meta-inference.

- Are conclusions warranted by the integrated findings?
- Are divergent findings incorporated rather than selectively ignored?
- Are claims of causality, generalization, transferability, mechanism, or experience appropriate to the design?

This domain also includes *ecological coherence.*

Ecological validity is particularly relevant in psychological science because psychological knowledge is frequently generated under varying degrees of experimental control. Krøjgaard (2001), drawing on the ecological psychology tradition, emphasizes that ecological validity should not become an abstract criticism of experimental research. Claims about ecological validity need to identify what features of ordinary contexts are absent and why those features matter to the phenomenon.

This insight is important for MMR. Qualitative inquiry does not automatically make quantitative results ecologically valid. Nor are naturalistic settings intrinsically superior to controlled ones. Instead, MMR can strengthen ecological understanding when complementary forms of evidence illuminate how a phenomenon varies between standardized measurement and lived or naturalistic contexts.

Thus the question is not simply:

*Do QUAL and QUAN agree?*

It is:

*What interpretation becomes defensible when convergence, complementarity, expansion, discordance, and contextual conditions are considered together?*

### 5.5 Domain 5: methodological transparency

The fifth domain is *methodological transparency*.

We define methodological transparency as:

*the extent to which readers can reconstruct the methodological reasoning, procedures, analytical decisions, points of integration, and inferential pathway through which a mixed methods study’s knowledge claims were produced.*

Transparency and coherence are distinct. A study may be internally coherent but poorly reported. Its coherence then remains inaccessible to readers.

A study can also be highly transparent but incoherent. Detailed reporting may reveal contradictions between questions, theory, design, methods, and inference.

This yields a simple conceptual distinction:

*Coherence asks whether the components belong together. Transparency asks whether readers can see why and how they belong together.*

Table 4 summarizes this distinction by illustrating how methodological coherence and transparency may vary independently while jointly shaping the assessability of MMR quality.

**Table 4:** Coherence and transparency as complementary dimensions of MMR quality.

Level of coherence	High methodological transparency	Low methodological transparency
High coherence	Strong/best-practice MMR: methodological relationships are defensible and critically assessable	Potentially sound research, but coherence is difficult to evaluate
Low coherence	Methodological mismatches are visible and can be critically evaluated	Weak and opaque MMR; neither the logic nor the relationships are adequately assessable

Transparency is particularly important in relation to existing concerns in psychology. Roberts and Allen (2019) note deficiencies concerning identification of MMR design, qualitative analysis, mixed methods questions, and integration. Likewise, Fàbregues et al. (2023) found generally reasonable reporting quality but persistent omissions concerning design detail, sampling, qualitative analysis, and presentation of integration.

Ketsman et al.’s (2025) findings extend the problem further: when a substantial proportion of studies that meet MMR criteria do not even identify themselves as mixed methods, the problem concerns not only reporting but methodological self-understanding.

Transparency is therefore both a reporting principle and an indicator of methodological literacy.

## 6 Vertical and horizontal coherence

The MMCTF can be further specified by distinguishing *vertical coherence* from *horizontal coherence.*

### 6.1 Vertical coherence

Vertical coherence concerns alignment through levels of the research architecture:

*phenomenon*

*↓*

*philosophical assumptions*

*↓*

*theory*

*↓*

*research questions*

*↓*

*design*

*↓*

*methods*

*↓*

*analyses*

*↓*

*integration*

*↓*

*meta-inference*

A theoretical construct operationalized through a psychological scale, for example, should maintain an intelligible relationship with how the construct is subsequently discussed in qualitative interviews and interpreted in the integrated findings.

Vertical incoherence can arise when conceptual definitions change silently between stages or when claims in the discussion exceed the operational meaning of variables and qualitative categories.

### 6.2 Horizontal coherence

Horizontal coherence concerns relationships between components working at comparable levels:

*QUAL ↔ QUAN.*

*qualitative findings ↔ quantitative findings.*

*emic perspective ↔ etic perspective.*

*individual case ↔ aggregate pattern.*

The point of integration is often where horizontal relationships become most visible. Importantly, horizontal coherence does *not* imply equivalence. A qualitative account of anxiety and a score on a validated anxiety measure are not interchangeable forms of information. Their value lies partly in their differences.

### 6.3 Connecting vertical and horizontal coherence

Vertical and horizontal coherence are analytically distinguishable but operate as interconnected dimensions of the overall research architecture. Figure 1 illustrates how these dimensions are brought together within the MMCTF, with points of integration linking qualitative and quantitative strands and meta-inference reconnecting these horizontal relationships to the broader vertical architecture. Whereas Bazeley (2024) conceptualizes coherence as a constitutive dimension of integration, the MMCTF extends the analytical scope by treating coherence as a higher-order property of the complete research architecture and by differentiating vertical, horizontal, and inferential coherence while positioning transparency as the condition through which these relationships can be critically evaluated.

**Figure 1:**
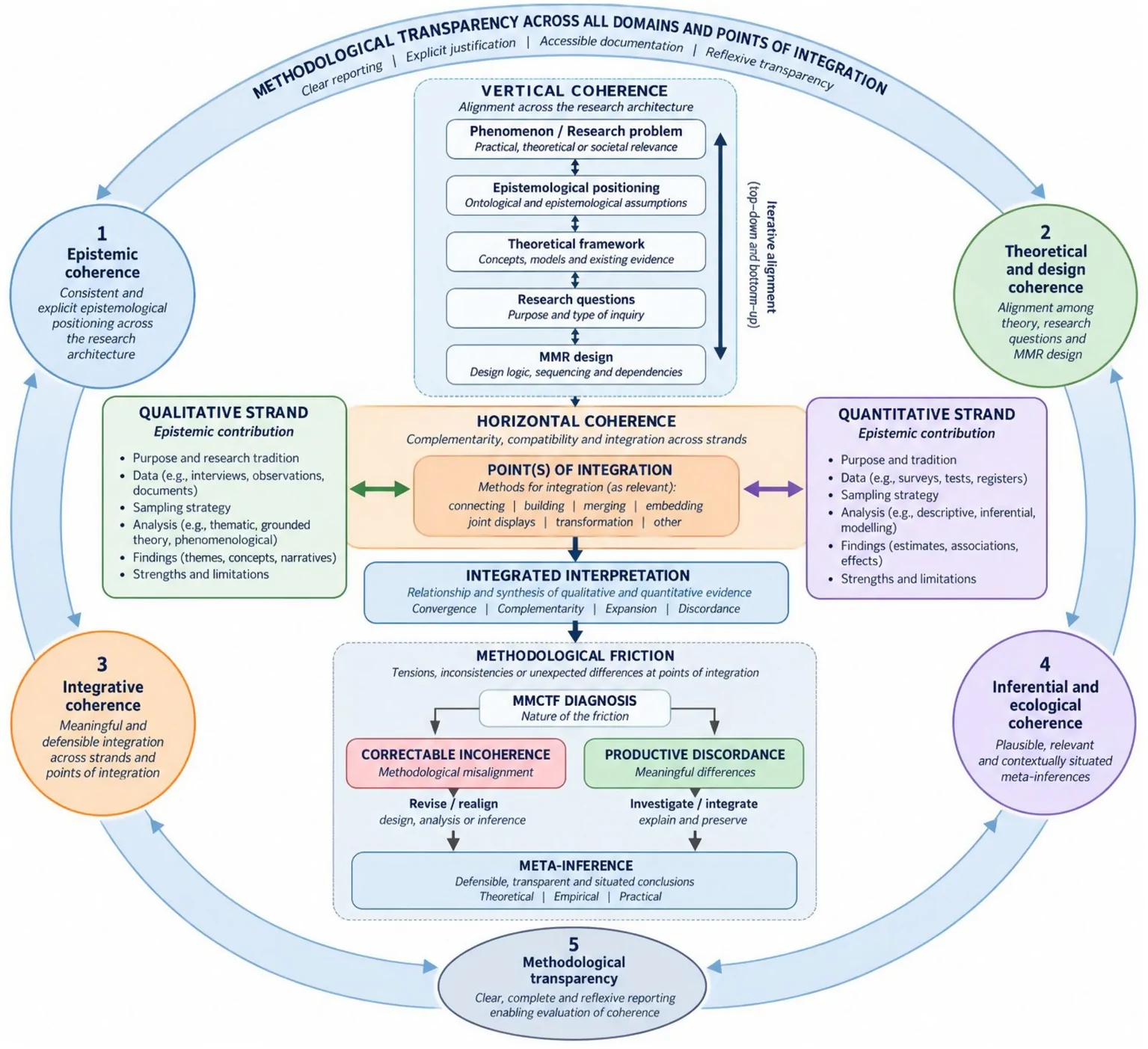
Proposed mixed methods coherence and transparency framework.

The framework conceptualizes coherence as a dynamic property of the overall mixed methods research architecture rather than as a linear research sequence. Vertical coherence concerns relationships among the research phenomenon, epistemological positioning, theory, research questions, and MMR design. Horizontal coherence concerns the relationship between qualitative and quantitative strands at one or multiple points of integration. Inferential coherence concerns the pathway from integrated evidence through methodological friction or fit toward defensible meta-inferences. The five MMCTF domains operate recursively across this architecture rather than as sequential stages. Methodological transparency spans all domains and integration points, enabling coherence to be critically evaluated. When methodological friction occurs, the framework distinguishes between correctable incoherence, requiring methodological reconsideration, and productive discordance, requiring investigation, integration, and transparent preservation in the resulting meta-inference.

## 7 From a research chain to a research architecture

Traditional research-methods representations commonly resemble linear chains:

*research question → data → analysis → conclusion.*

Such representations can be useful pedagogically but are often inadequate for complex MMR. Sequential designs contain feedback loops. Exploratory findings can generate instruments. Quantitative findings can reshape qualitative sampling. Emergent designs may change in response to unexpected results. Contradictory findings may trigger reanalysis of earlier data. Joint displays can produce new questions requiring return to original datasets. MMR should therefore be conceptualized as a *research architecture* rather than merely a research sequence. This distinction has practical implications.

An architecture concerns both components and relationships. A structurally impressive building can fail if connections between components are unsound. Likewise, an MMR project may contain advanced statistical analysis, rich qualitative data, several theoretical perspectives, and multiple publications while still lacking an intelligible logic connecting them. This is why methodological sophistication and methodological coherence should not be treated as synonymous.

The MMCTF therefore does not prescribe one optimal architecture. Instead, it asks whether the architecture chosen is defensible for the phenomenon under investigation.

### 7.1 Best-practice questions derived from the MMCTF

The conceptual domains can be translated into practical questions relevant to researchers, reviewers, editors, and doctoral candidates. Table 5 operationalizes the five MMCTF domains by summarizing key questions, indicators, and illustrative examples that can guide this evaluation.

**Table 5:** The five domains of the mixed methods coherence and transparency framework (MMCTF).

MMCTF domain	Key questions for researchers (What to consider)	Illustrative indicators (Signs of coherence)	Example (psychological research)
1. Epistemic coherence *Consistent and explicit epistemological positioning across the research architecture*	1. What kind of knowledge claims are made in the qualitative and quantitative components? 2. Are the epistemological assumptions compatible or are differences explicitly acknowledged and justified? 3. Are these assumptions consistently reflected in the research questions, methods, analysis and interpretation? 4. How do the components contribute to addressing the research problem?	• Explicit epistemological rationale for using multiple methods • Consistent alignment between epistemology, methods and claims • Justification of epistemological differences, where present • Complementary contributions to the overall knowledge claim	A study of adolescent mental health combines statistical estimates of symptom prevalence (positivist/critical realist assumptions) with qualitative accounts of lived experience (constructivist assumptions), with explicit justification of how different forms of knowledge contribute to the research problem.
2. Theoretical and design coherence *Alignment among theory, research questions and MMR design*	1. Is there a clear theoretical framework guiding the overall study? 2. Are the research questions aligned with the theoretical framework and the chosen MMR design? 3. Are the qualitative and quantitative components purposively designed and sequenced (where relevant)? 4. Are dependencies between components explicitly justified?	• Clear theoretical framework and rationale for MMR design. • Explicit alignment between research questions and methods. • Justified sampling, sequencing and prioritization. • Design choices consistent with the intended integration and knowledge claims	A mixed methods study on psychotherapy outcomes is guided by a theoretical model of change, with an explanatory sequential design where initial quantitative results inform the sampling and focus of subsequent qualitative interviews.
3. Integrative coherence *Meaningful and defensible integration across strands and points of integration*	1. Where and how do the qualitative and quantitative components interact? 2. Which integration strategy (e.g., connecting, building, merging, embedding, joint display, transformation) is used, and why? 3. Are the integration procedures consistent with the design and research questions? 4. Are differences, complementarities or contradictions between findings explicitly examined and incorporated?	• Explicit point(s) and method(s) of integration. • Integration strategy consistent with design and purpose. • Systematic comparison, combination or transformation of findings. • Transparent handling of convergence, complementarity, expansion and discordance	Quantitative and qualitative findings on an anxiety intervention are integrated through a joint display that links changes in symptom scores with participants’ accounts of what was helpful or less helpful, including cases with divergent experiences.
4. Inferential and ecological coherence *Plausible, relevant and contextually situated meta-inferences*	1. What contextual or person-level conditions qualify the integrated findings? 2. Do qualitative findings suggest theoretically defensible moderators, mechanisms or boundary conditions? 3. Can these propositions legitimately inform further quantitative analysis, or should they instead bound the interpretation?4. Does the meta-inference specify for whom, under what conditions, and in which contexts the conclusion is expected to hold?	• Explicit consideration of contextual, temporal and population boundaries. • Qualitative evidence informs (where appropriate) moderators, mechanisms or subgroup analyses. • Clear justification for additional analyses or for bounding the inference without further testing. • Meta-inference specifies scope, conditions and limitations	Interviews indicate that the effect of a mindfulness-based intervention varies by social support and ability to integrate the practice into daily life. These insights are used to examine support as a moderator in quantitative analyses and to bound the interpretation of the overall effect.
5. Methodological transparency *Clear, complete and reflexive reporting enabling evaluation of coherence*	1. Are the rationale for using MMR, the design, methods, analysis and integration procedures clearly reported? 2. Are decisions, adaptations and analytical uncertainties documented? 3. Is the role of researchers, including use of tools such as AI, transparently described? 4. Is there sufficient information for others to critically evaluate the coherence of the study?	• Transparent and complete reporting of methodological decisions. • Documentation of adaptations and unresolved issues. • Reflexive account of researcher positionality and use of AI tools (where relevant). • Sufficient detail to allow critical appraisal and potential replication	The study provides a transparent account of how qualitative and quantitative data were collected, analyzed and integrated, including the use of AI-assisted qualitative coding, with documentation of prompts, analytical adjustments and human validation.

MMR, mixed methods research; AI, artificial intelligence.

These questions should not be interpreted as a rigid checklist or scoring system. Such a use could undermine the methodological pluralism the framework seeks to preserve. Rather, the questions function as *conceptual prompts* for identifying relationships that require methodological justification.

## 8 Generative AI as a catalyst for new points of integration

The increasing use of artificial intelligence in research introduces new methodological possibilities as well as new challenges for mixed methods coherence and transparency. AI may support analytical processes within qualitative and quantitative strands, but it may also mediate the relationships through which evidence is transformed, integrated, and interpreted. This section therefore examines how generative and predictive AI may create new points of integration within MMR and considers their implications for the MMCTF.

### 8.1 AI-mediated mixed methods research

Artificial intelligence should not be conceptualized merely as an additional analytical tool within an otherwise unchanged mixed methods research process. When AI participates in coding, classification, summarization, pattern identification, comparison of qualitative and quantitative findings, construction of joint displays, exploration of discordant cases, or formulation of candidate integrative interpretations, it may alter the analytical relationships through which evidence is generated, transformed, and integrated. AI can therefore create additional *human – AI - data interfaces* and potentially new points of integration within an MMR research architecture.

This development extends the conventional understanding of integration. In human-led MMR, qualitative and quantitative components may be connected, built upon, merged, embedded, transformed, or integrated during interpretation. AI-mediated MMR may introduce additional analytical interfaces through which evidence is selected, transformed, summarized, classified, compared, privileged, or excluded. The methodological question therefore becomes not only *where and how were qualitative and quantitative components integrated? but also where did AI enter the analytical architecture, what function did it perform, how did it influence integration, and how was its contribution evaluated by human researchers?*

We use the following representation to capture this expanded architecture:

*QUAL evidence ↔ AI-mediated analytical interface ↔ QUAN evidence ↔ human interpretation.*

This representation does not imply that AI must occupy a position between qualitative and quantitative evidence, nor that every use of AI constitutes integration. Rather, it highlights that AI can enter at different stages and alter the number, location, and character of methodological interfaces that require justification and transparency.

### 8.2 AI as a catalyst for new points of integration

AI-mediated integration may occur at multiple stages of an MMR design. In an exploratory sequence, qualitative evidence could contribute to identifying constructs, categories, or linguistic patterns that are subsequently operationalized quantitatively with AI-supported analytical procedures:

*QUAL → AI-supported pattern identification → QUAN operationalization.*

A reverse pathway is also possible. Predictive or pattern-detection methods may identify heterogeneous trajectories, subgroups, anomalies, or unexpected cases that subsequently guide purposive qualitative sampling:

*QUAN → AI-supported subgroup or anomaly identification → QUAL follow-up.*

AI may also enter directly at an integrative stage:

*QUAL + QUAN → AI-supported comparison → human evaluation and interpretation → meta-inference.*

For example, in a psychological intervention study, quantitative symptom trajectories might be combined with qualitative interviews concerning participants’ experiences of treatment. AI could support the identification of candidate qualitative patterns associated with different quantitative trajectories or generate possible explanations for discordant cases. Such outputs could help researchers identify relationships requiring closer examination, but they would not constitute meta-inferences by themselves. Researchers would need to return to the qualitative material, quantitative results, theoretical framework, and contextual evidence to determine whether the proposed relationship is empirically and methodologically defensible.

AI may therefore increase the number and complexity of points of integration in MMR. However, *every consequential AI-mediated integration point simultaneously increases the requirement for methodological justification, transparency, and human evaluation*.

### 8.3 Distinguishing generative AI from predictive AI in mixed methods research

Artificial intelligence encompasses analytically distinct technologies that should not be treated as methodologically interchangeable. Of particular relevance to contemporary MMR is the distinction between generative AI, including large language models (LLMs), and predictive AI based primarily on machine-learning approaches. Although both may contribute to the same research architecture, they typically perform different analytical functions and consequently create different requirements for coherence, integration, validation, and transparency.

Generative AI and LLMs operate primarily through the generation, transformation, comparison, and synthesis of content. Within MMR, they may support summarization, candidate coding or categorization, comparison across datasets, exploration of discordant cases, generation of candidate explanations, construction of preliminary joint displays, or iterative analytical dialogue with researchers. Their methodological significance lies particularly in their potential to influence interpretive and integrative processes. Because generated outputs may appear linguistically persuasive even when insufficiently grounded in the empirical material, provenance, reflexivity, empirical verification, and human interpretive accountability become particularly consequential.

Predictive AI and machine-learning approaches, by contrast, are typically oriented toward classification, prediction, clustering, estimation, or pattern detection in structured or high-dimensional data. Within psychological research, such approaches may identify risk profiles, latent subgroups, behavioral patterns, or individual outcome trajectories. Their integration into MMR may occur when qualitative evidence is used to explain model outputs, investigate unexpected classifications, contextualize identified subgroups, inform interpretation of predictive features, or examine mechanisms underlying observed patterns. Conversely, qualitative findings may generate theoretically grounded distinctions that inform subsequent quantitative or machine-learning analyses.

The distinction is therefore both methodological and epistemic. GenAI may function as an intermediary within interpretive or integrative processes, whereas predictive AI may constitute part of the quantitative evidence that subsequently requires contextualization or explanation. However, these categories are not mutually exclusive. LLMs are themselves machine-learning systems and can perform classification and predictive tasks, while contemporary research architectures may combine predictive and generative systems. The distinction proposed here is therefore functional rather than ontological: AI should be characterized according to the analytical function it performs within the particular research architecture.

This functional distinction guards against two forms of inferential slippage:

*Predictive output is not explanation, and generative output is not interpretation by default.*

In both cases, the transition from computational output to substantive knowledge claim requires theoretical justification, integration with relevant evidence, and human epistemic accountability.

### 8.4 Human - AI division of analytical labour and interpretive presence

We use the term *human–AI division of analytical labour* to describe the explicit allocation of computational or analytical-support functions, interpretive authority, and epistemic responsibility between human researchers and AI systems. The concept does not presume that AI and human researchers perform epistemically equivalent forms of analysis. Rather, it requires researchers to specify what an AI system contributes, how that contribution influences subsequent analytical decisions, and what evidential status is assigned to its outputs.

This distinction is important given current disagreement about whether LLM-generated outputs should themselves be characterized as qualitative analysis (Friese, 2026; Nguyen and Welch, 2026). Within the MMCTF, AI-generated codes, categories, summaries, comparisons, classifications, or explanations are therefore treated as candidate analytical outputs whose methodological status depends on the research design, epistemological position, analytical function, human engagement with the underlying evidence, and subsequent evaluation. Human researchers remain responsible for the interpretations and knowledge claims ultimately advanced by the study.

AI involvement also creates what we term *interpretive presence*. GenAI does not possess researcher positionality or lived engagement with participants, yet AI-generated categories, summaries, comparisons, or explanations may shape subsequent human decisions and thereby influence the interpretive pathway through which findings and meta-inferences are produced. The relevant methodological issue is therefore not whether an AI system “interprets” in the same sense as a human researcher, but whether and how its outputs influence human interpretation.

From an MMCTF perspective, this influence should remain visible. Researchers should be able to distinguish, where methodologically consequential, among interpretations originating primarily from human engagement with the evidence, candidate propositions generated by AI, and interpretations emerging through iterative human - AI interaction (Friese, 2026).

This distinction also clarifies our use of *symbiotic intelligence*. We use this term more narrowly to describe research configurations in which human methodological judgement and AI-supported analytical capabilities perform complementary functions while human researchers retain epistemic responsibility for the resulting knowledge claims. Symbiotic intelligence is therefore not synonymous with the human - AI division of analytical labour. The former describes a particular collaborative configuration; the latter specifies the methodological allocation of analytical functions and epistemic responsibility within that configuration.

### 8.5 Contested epistemic authority, bias, and human validation

The methodological role of GenAI remains contested, particularly in qualitative and interpretive research. Emerging scholarship ranges from structured proposals for AI-assisted analytical dialogue to fundamental concerns about whether probabilistically generated text can legitimately participate in qualitative meaning-making. Friese (2026), for example, proposes a conversational framework involving iterative questioning, synthesis, and reflexive interpretation. Nguyen and Welch (2026), by contrast, caution against conflating the probabilistic generation of synthetic text with human interpretation of social meaning. Greenhalgh (2026) advances a more differentiated position in which a central concern is whether AI use displaces, obscures, or constrains researchers’ reflexive engagement with the data.

The MMCTF does not require this debate to be resolved through either unconditional adoption or categorical prohibition. Instead, the disagreement illustrates why epistemic coherence becomes especially consequential when the epistemic status of the analytical technology itself is contested. An AI-supported procedure that is defensible within one methodological configuration cannot automatically be transferred to another. The relevant question is not simply *Can AI perform this task?* but *Is the role assigned to AI epistemically and methodologically coherent with this particular research architecture?*

AI-mediated analysis also introduces additional pathways for bias. Model outputs may reproduce biases embedded in training data, privilege linguistically dominant interpretations, flatten ambiguity, overstate patterns, or generate apparently coherent explanations that remain insufficiently grounded in the empirical evidence. These risks may become particularly consequential when AI-generated outputs influence how differences or discordance between methodological strands are interpreted.

Human evaluation should therefore extend beyond judging whether an AI output appears plausible. Researchers should examine empirical grounding, theoretical consistency, sensitivity to alternative interpretations, treatment of negative and discordant cases, contextual adequacy, and consequences for the resulting meta-inference. When AI proposes an explanation for a QUAL - QUAN discrepancy, for example, researchers should return to the underlying evidence rather than accepting the model’s integrative fluency as evidence of methodological fit.

Accordingly:

*AI-generated coherence should not be mistaken for methodological coherence.*

### 8.6 AI-mediated transparency and analytical audit trails

AI-mediated research requires a distinction between disclosure and methodological transparency. Disclosure establishes that AI was used; methodological transparency establishes how its use influenced the production, transformation, integration, and interpretation of evidence. Emerging reporting approaches such as TROUT-AI (Jones, 2025) reflect this concern. In MMR, transparency additionally needs to encompass points of integration and meta-inference.

Where AI materially contributes to analysis or integration, researchers should provide an AI-mediated analytical audit trail that makes consequential human–AI interactions sufficiently visible to reconstruct AI’s influence on the pathway from evidence to knowledge claim. This should identify, where relevant, the system/model, purpose and stage of use, data involved, human–AI division of analytical labour, consequential interactions, use or rejection of outputs, and human evaluation.

The underlying principle is proportional transparency: the greater the epistemic and analytical influence of AI on findings, integration, and meta-inference, the greater the requirement for methodological documentation and human evaluation. Minor administrative or linguistic assistance therefore does not require the same documentation as AI-supported coding, classification, integration, or interpretation. Methodological relevance, rather than prompt volume, should determine what is reported.

Transparency nevertheless remains distinct from coherence: transparency is not evidence of coherence; it is a precondition for evaluating coherence.

### 8.7 AI as a stress test of methodological coherence

AI can also function as a stress test of methodological coherence. If relationships among the research problem, epistemological assumptions, theory, research questions, design, methods, and integration strategy are poorly articulated, adding technically sophisticated AI analyses will not resolve the underlying methodological problem and may instead increase complexity and opacity.

The preferred direction of methodological reasoning therefore remains:

*research problem → epistemic and theoretical rationale → MMR purpose and design → integration need → analytical procedure → technology selection*.

rather than:

*available AI capability → analytical application → retrospective methodological justification.*

The MMCTF consequently treats technology selection as downstream from methodological reasoning. AI should enter the research architecture because it serves a specified methodological or analytical function, rather than becoming the starting point around which methodological justification is subsequently constructed.

### 8.8 AI-mediated extension of the MMCTF

The implications of AI for MMR are best understood as an extension of the MMCTF rather than as a separate methodological paradigm. Figure 2 illustrates how conventional qualitative and quantitative inquiry may interact with generative and predictive AI within a common research architecture. The research phenomenon, epistemological positioning, theoretical framework, research questions, and MMR design remain methodological anchors, while AI may enter at different analytical stages and points of integration.

AI-supported research is therefore not conceptualized as an alternative to conventional MMR, nor does the inclusion of AI create methodological coherence by itself. Instead, different AI functions introduce additional interfaces that must be evaluated through the same five MMCTF domains.

Table 6 translates the five MMCTF domains into AI-specific coherence questions. These questions are not intended as a separate AI appraisal framework but as an extension of the same epistemic, theoretical/design, integrative, inferential/ecological, and transparency requirements applied throughout the MMCTF.

**Table 6:** MMCTF - AI-mediated coherence question.

MMCTF domain	AI-mediated coherence question
Epistemic coherence	Is the epistemic authority assigned to AI compatible with the methodological tradition and intended knowledge claims?
Theoretical and design coherence	Was AI selected because it serves the research question, theoretical rationale, and design rather than primarily because the technology was available?
Integrative coherence	Where does AI create or modify points of QUAL–QUAN integration, and how does it transform, compare, select, or privilege evidence?
Inferential and ecological coherence	Are AI-supported outputs empirically grounded and sufficiently sensitive to context, discordance, subgroup variation, and boundary conditions?
Methodological transparency	Can readers reconstruct the human - AI division of analytical labour and evaluate how AI influenced analytical decisions, findings, integration, and meta-inferences?

Whereas Figure 1 presents the general architecture of the MMCTF, Figure 2 illustrates its extension when generative or predictive AI becomes part of the analytical and integrative process. Generative AI/LLMs may contribute to transformation, comparison, synthesis, and candidate integrative interpretations, whereas predictive AI/machine learning may contribute to classification, prediction, pattern detection, and subgroup identification. Neither form of computational output constitutes explanation, interpretation, or meta-inference by itself. Its contribution must be integrated with relevant qualitative, quantitative, theoretical, and contextual evidence and evaluated through the five MMCTF domains. Methodological transparency and human epistemic accountability span the complete architecture.

**Figure 2:**
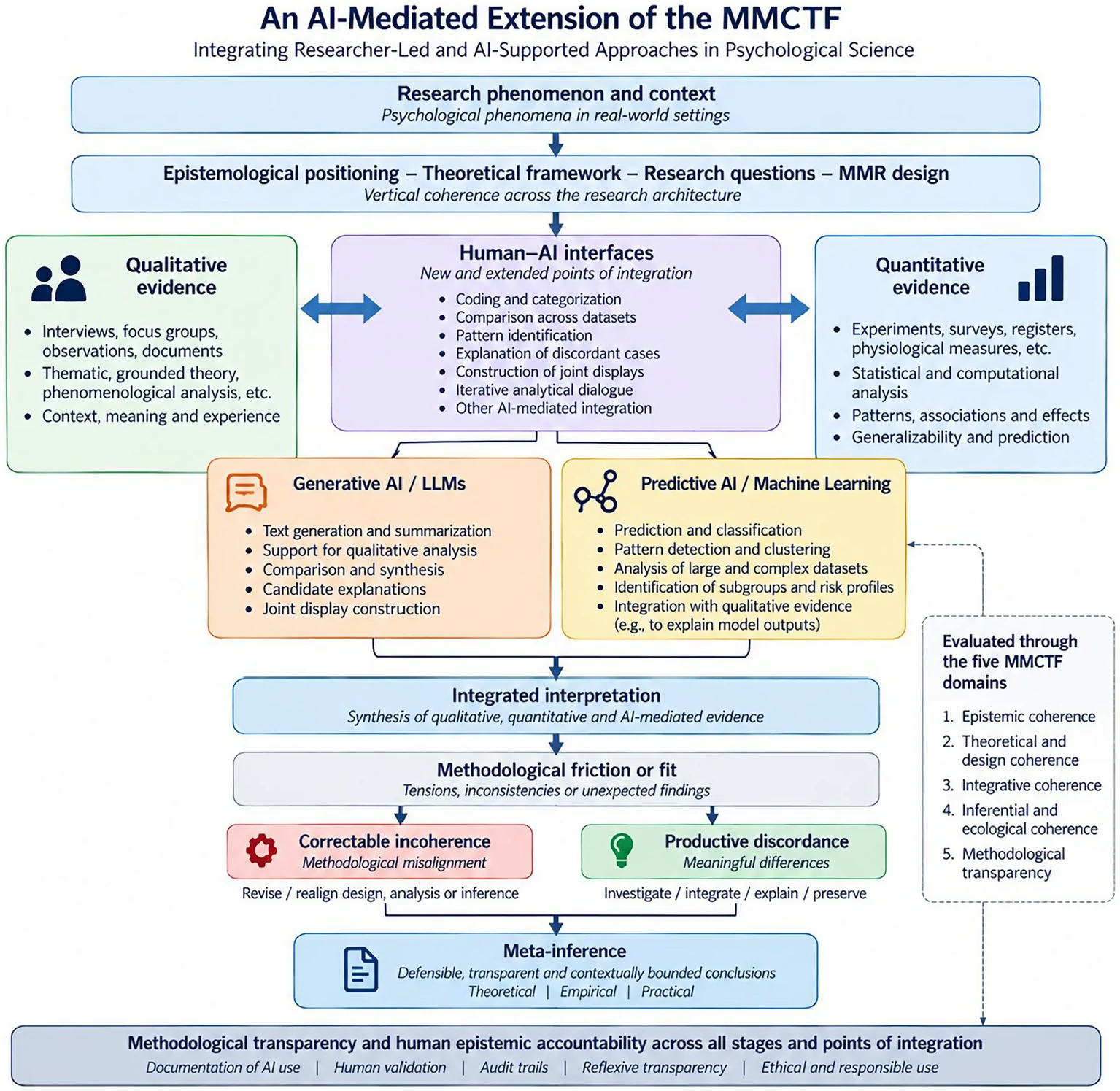
AI-mediated extension of mixed methods coherence and transparency framework (MMCTF). AI-supported analytical approaches may enter a mixed methods research architecture at different stages and create additional human–AI–data interfaces and points of integration. Generative AI/LLMs and predictive AI/machine learning perform different analytical functions and require different forms of integration, validation, and transparency. Their contribution must be evaluated through the five MMCTF domains.

Figure 2 also focuses on complex psychological phenomena in real-world settings. This distinction is particularly relevant in psychological science because AI-supported research may simultaneously involve computational prediction and interpretation of context-sensitive human experience. For example, machine-learning models may identify patterns of psychological risk or heterogeneous treatment trajectories, while qualitative inquiry examines how those patterns are experienced and situated within participants’ social and personal contexts. GenAI may subsequently support comparison across these forms of evidence. Such a configuration creates multiple points of integration but also multiple opportunities for epistemic slippage, particularly if predictive patterns are treated as explanations or AI-generated syntheses are treated as interpretations without adequate human evaluation.

## 9 Implications for psychological science

The MMCTF has particular relevance to psychological science because many psychological phenomena span the boundaries between measurement and meaning, general patterns and individual experience, and experimental control and contextual complexity. Constructs such as self-efficacy, anxiety, motivation, identity, perceived control, and wellbeing may be represented quantitatively through psychometric measures while also requiring attention to how individuals experience and interpret them. Similarly, average associations or intervention effects may conceal psychologically meaningful heterogeneity across individuals, subgroups, contexts, or trajectories. MMR can bring these nomothetic and idiographic forms of evidence into productive relationship, but doing so requires more than their inclusion within the same study: their epistemic functions, points of integration, and contribution to the resulting meta-inference must form a defensible research architecture.

These considerations become increasingly consequential as psychological research incorporates computational and AI-supported approaches. Predictive AI may identify patterns, risk profiles, or heterogeneous trajectories, while qualitative inquiry can contextualize how such patterns are experienced and what they mean within participants’ lives; GenAI may additionally mediate analytical or integrative processes. Such methodological plurality creates opportunities for richer explanation but also increases the risk of epistemic slippage, decontextualization, and performative complexity. Psychological science therefore provides a particularly relevant domain for shifting the methodological question from whether qualitative and quantitative methods have been mixed to whether the complete research architecture is coherent, transparent, and capable of supporting defensible meta-inferences.

Although developed and illustrated here in relation to psychological science, the underlying coherence principles are potentially transferable to other fields in which qualitatively and quantitatively derived evidence is integrated; examining such transferability represents an important direction for future evaluation of the MMCTF.

## 10 Implications for researchers, reviewers, and editors

The MMCTF has practical implications for how MMR is designed, conducted, evaluated, and communicated. These implications concern not only individual researchers but also the institutional and scholarly environments in which methodological decisions are made. The following subsections consider pressures that may shape methodological choices and outline implications for researchers, reviewers, and editors.

### 10.1 Journal, funding, and doctoral pressures

Such pressures may arise in different forms. Researchers may perceive expectations from journals, funding environments, doctoral programmes, collaborators, or disciplinary trends to demonstrate methodological breadth. These expectations are not inherently problematic and may encourage valuable methodological pluralism. The coherence problem arises when the external rationale for adopting MMR becomes stronger than the internal methodological rationale generated by the research question.

External rationale for MMR ≠ internal methodological rationale for MMR.

The MMCTF can therefore function as a form of methodological resistance to performative complexity. Rather than rewarding the accumulation of methods, datasets, integration techniques, or AI-supported analytical procedures, it requires researchers to justify the contribution and relationship of each component to the research architecture and resulting meta-inference.

The same principle becomes increasingly important as AI enters research practice. The availability of GenAI and machine-learning tools may create a parallel form of technological performativity, in which AI is incorporated because of novelty, accessibility, perceived sophistication, or expectations of innovation rather than because it addresses a clearly specified analytical or integrative need. Adding AI to an MMR design does not make the study more advanced, just as adding a qualitative component to a quantitative study does not necessarily make it meaningfully mixed.

The MMCTF therefore reverses a technology-first logic. The preferred sequence is:

*research problem → epistemic and theoretical rationale → MMR purpose and design → integration need → analytical procedure → technology selection.*

Technology should enter the architecture because it serves a methodological function, rather than becoming the starting point around which a methodological rationale is subsequently constructed.

The same caution applies to the MMCTF itself. The framework is not intended as a compliance checklist in which researchers demonstrate the presence of five domains. Such use would reproduce the performative logic the framework is intended to challenge. Rather, the domains function as critical questions about relationships within the research architecture. Coherence is not demonstrated by checking boxes but through an explicit and defensible account of why the methodological components belong together and how their relationships support the resulting meta-inference.

### 10.2 Implications for researchers: designing for coherence

Researchers should begin by articulating why MMR is needed.

The question should not be:

*How can I add a qualitative component to my quantitative study?*

or:

*How can I validate my qualitative findings statistically?*

Instead:

*What is insufficiently understood about this phenomenon through one methodological perspective, and how can purposeful integration address that insufficiency?*

Researchers should then identify the purpose of mixing, theoretical drive, points of integration, anticipated relationship among findings, and logic of meta-inference. This is especially important when designs become emergent or complex.

### 10.3 Implications for reviewers: evaluating coherence and integration

Reviewers should not equate methodological credibility with correct use of design terminology. A manuscript may accurately label itself “explanatory sequential” yet demonstrate little meaningful explanation. Conversely, a methodologically sophisticated study may use a hybrid or interactive design that does not fit neatly into a textbook label.

Reviewers should therefore examine:

- the rationale for MMR
- the relationship among research questions and components
- the actual points of integration
- the added value produced through mixing
- the justification of the meta-inferences

The MMCTF can provide a conceptual structure for this evaluation without becoming a mechanical checklist.

### 10.4 Implications for editors: supporting methodological transparency

Journal editors influence methodological quality through expectations for reporting and peer review. Clearer requirements that authors identify their mixed methods architecture, purpose of mixing, integration mechanisms, and meta-inferences could address some of the reporting limitations identified in the literature.

The problem documented by Ketsman et al. (2025)—where many studies fulfilling MMR criteria do not identify themselves as mixed methods at all - also has implications for evidence synthesis. Poor methodological indexing and reporting make it difficult for systematic reviewers to retrieve, classify, and evaluate relevant studies. Transparency therefore has consequences beyond individual publications.

## 11 Discussion

This conceptual analysis has sought to move the discussion of mixed methods quality beyond integration without moving away from integration. Integration remains a defining feature of MMR and a necessary condition for distinguishing mixed methods inquiry from the parallel use of qualitative and quantitative methods. Foundational work has substantially advanced understanding of how integration can occur through connecting, building, merging, embedding, and integration at the level of interpretation and reporting (Fetters et al., 2013; Schoonenboom and Johnson, 2017). Subsequent scholarship has further developed joint displays, successive integration, qualitatively oriented mixed methods, and the development of meta-inferences (McCrudden et al., 2021; Poth and Shannon-Baker, 2022; Schoonenboom, 2022, 2023).

More recent developments have shifted attention from the *presence* of integration toward its *quality*. The Mixed Methods Integration Quality Framework (MMIQF), for example, synthesizes criteria for evaluating the quality of integration across the research process (Fàbregues et al., 2024), while recent work on meta-inference distinguishes among confirmed, discordant, expanded, and complementary relationships between findings (Younas et al., 2025). Bazeley (2024) similarly conceptualizes integration as a dynamic and interdependent process rather than a discrete procedural event. Together, these developments represent an important maturation of MMR methodology.

The MMCTF builds on, rather than replaces, these advances. Its distinctive contribution is to shift the analytical lens from integration alone toward the research architecture within which integration occurs. Whereas integration quality concerns the integrity of the processes through which qualitative and quantitative components are brought into relationship, coherence concerns whether the phenomenon under investigation, epistemological assumptions, theoretical framework, research questions, MMR purpose and design, methodological traditions, analytical procedures, integration mechanisms, and resulting meta-inferences form an explicit and defensible whole. In this sense, integration is necessary but insufficient for coherence.

This distinction has practical consequences. A point of integration may be technically identifiable while remaining theoretically weakly justified. A joint display may juxtapose qualitative and quantitative evidence without producing an inferentially meaningful relationship. A sequential design may connect two phases procedurally without establishing conceptual continuity between them. Conversely, methodological tension or divergent findings do not necessarily indicate incoherence. A coherent MMR study may generate confirmation, complementarity, expansion, or theoretically meaningful discordance (Younas et al., 2025). Coherence should therefore not be confused with convergence. The relevant criterion is whether the relationship between different forms of evidence is explicitly investigated, methodologically justified, and incorporated into a defensible meta-inference.

The MMCTF consequently conceptualizes coherence as a higher-order property of the research architecture. Alignment concerns relationships between particular components - for example, between research questions and methods. Integration concerns the interfaces through which qualitative and quantitative components are brought into relationship. Coherence concerns whether these alignments and interfaces collectively constitute a defensible research architecture. Transparency, in turn, determines whether this architecture can be critically examined. The five domains of the MMCTF organize these relationships across epistemic, theoretical and design, integrative, inferential and ecological, and transparency dimensions.

### 11.1 The contribution of epistemic coherence

The first contribution of the MMCTF is to foreground the epistemic relationships underlying MMR without reviving the paradigm wars. Methodological pluralism does not require philosophical neutrality or the elimination of epistemological difference. Researchers may work from pragmatic, dialectical, transformative, realist, qualitatively driven, quantitatively driven, or other positions. The relevant question is whether the epistemological commitments associated with different components of the study and the knowledge claims derived from them are made sufficiently explicit and brought into a defensible relationship.

This is particularly consequential in psychological science, where MMR may bring nomothetic forms of evidence - such as psychometric measures, population-level associations, experimental effects, or predictive models - into relationship with idiographic and interpretive evidence concerning lived experience, meaning, context, and individual variation. These forms of evidence need not share identical epistemological assumptions to contribute to a coherent study. Indeed, their differences may constitute part of the methodological rationale for mixing. The requirement is therefore not epistemological homogenization but epistemically defensible pluralism.

This perspective also guards against an instrumental conception of MMR in which QUAL and QUAN are treated merely as different data formats. Qualitative traditions such as phenomenology and grounded theory make distinctive epistemic and methodological contributions that should not be erased at the point of integration. Epistemic coherence therefore requires attention not only to *what* evidence is integrated but also to the conditions under which different forms of evidence can legitimately contribute to the resulting knowledge claims.

### 11.2 The contribution of theoretical and design coherence

The second contribution concerns the relationship among theory, research questions, and MMR design. Methodological complexity can encourage theoretical eclecticism because different components may appear to require different conceptual lenses. Such pluralism may be productive, but it can also fragment the research architecture if the function of each theoretical perspective remains unclear.

The MMCTF therefore does not prescribe theoretical uniformity. Instead, it asks whether the theoretical framework or frameworks perform identifiable functions and whether these functions remain connected to the research problem, research questions, methodological components, integration strategy, and meta-inference. Multiple theoretical perspectives can consequently be coherent when their relationship is explicit and analytically productive.

The same principle applies to MMR design. Established design typologies provide important methodological scaffolding, but design labels should not substitute for methodological reasoning. Identifying a study as convergent, explanatory sequential, exploratory sequential, embedded, or another design does not itself establish coherence. The design should follow from the research problem and intended purpose of mixing. This positions methodological necessity before methodological complexity: the relevant question is not how sophisticated the design appears, but why the research problem requires the methodological architecture selected.

### 11.3 The contribution of integrative coherence

The third contribution is to conceptualize a *point of integration* not only as a location in the research process but as a relationship requiring methodological explanation. Researchers should therefore move beyond statements that integration occurred “during analysis” or “during interpretation” and specify what was connected, compared, transformed, merged, embedded, or otherwise brought into relationship; why this particular integration was necessary; and what became inferentially possible because the relationship was established.

This extends descriptive integration toward explanatory integration. Importantly, integrative coherence does not imply that integration must produce agreement. Recent work on meta-inference reinforces that integration can produce confirmed, complementary, expanded, or discordant relationships among findings (Younas et al., 2025). Discordance may reveal differences in constructs, levels of analysis, temporal processes, contexts, subgroups, or participant experiences that would remain obscured within either strand alone.

The MMCTF therefore distinguishes correctable incoherence from productive discordance. Correctable incoherence occurs when methodological friction reflects an unjustified mismatch among research questions, theoretical assumptions, design, methods, analysis, or inference and consequently calls for methodological revision. Productive discordance occurs when appropriately conducted methodological components generate different but defensible insights into the phenomenon. In the latter case, the appropriate response is not to force convergence but to investigate, explain, integrate, and where theoretically warranted preserve the difference within the meta-inference.

### 11.4 The contribution of inferential and ecological coherence

The fourth contribution concerns what happens after integration. Mixed methods findings do not interpret themselves, and the presence of integrated evidence does not guarantee a defensible meta-inference. Inferential coherence therefore asks whether the conclusions drawn from integrated evidence are warranted by the qualitative and quantitative components and by the relationship established between them.

Ecological coherence extends this requirement by asking whether the resulting meta-inference remains plausible and appropriately bounded when situated within the populations, contexts, settings, and conditions in which the psychological phenomenon occurs. In this sense, ecological coherence should not be equated simply with ecological validity or naturalistic research. Rather, it concerns the correspondence between contextual evidence and the defensible boundaries of the meta-inference.

This creates a potentially important bridge between qualitative contextual knowledge and quantitative analysis. Qualitative findings may identify theoretically plausible moderators, mechanisms, subgroup differences, contextual boundary conditions, or alternative construct specifications that warrant further quantitative examination. Conversely, quantitative heterogeneity may guide qualitative follow-up toward particular cases, groups, trajectories, or contexts. However, the MMCTF does not imply that every qualitative observation should be converted into a quantitative moderator or tested *post hoc*. Additional analyses require theoretical justification, adequate measurement and statistical support, and transparent differentiation between confirmatory and exploratory analysis. Where these conditions are absent, contextual evidence can still strengthen ecological coherence by bounding the scope of the meta-inference rather than generating additional statistical tests.

Thus, statistical generalizability and ecological coherence address related but different questions. Statistical generalizability concerns how broadly an observed quantitative pattern may extend; ecological coherence asks whether the scope of the *integrated* conclusion is consistent with what the quantitative patterns and contextual evidence together permit researchers to claim.

### 11.5 The contribution of methodological transparency

The fifth contribution is to distinguish methodological transparency from coherence itself. Transparency is not evidence of coherence; it is a precondition for evaluating coherence. A study may possess an internally coherent methodological architecture while reporting it inadequately. Conversely, detailed reporting does not transform an incoherent design into a coherent one. Transparency and coherence should therefore be understood as analytically distinct but mutually dependent dimensions of methodological quality.

Recent methodological reviews reinforce the importance of this distinction. Reviews in educational psychology and mental health research demonstrate substantial variation in the reporting of MMR rationales, design choices, integration procedures, and methodological rigor (McCrudden et al., 2025; Demkowicz et al., 2025). Such findings should not automatically be interpreted as evidence of incoherence, because reporting limitations do not reveal the complete underlying research process. They do, however, demonstrate a fundamental evaluative problem: when methodological relationships are insufficiently visible, their coherence cannot be adequately assessed.

This requirement becomes more consequential as AI enters the analytical and integrative architecture of MMR. GenAI/LLMs and predictive AI/machine-learning approaches can introduce additional human – AI - data interfaces and potentially new points of integration. Their functions should not be conflated. Predictive AI may contribute classifications, probabilities, trajectories, risk profiles, or patterns that subsequently require contextualization and integration, whereas GenAI may contribute candidate codes, summaries, comparisons, explanations, or integrative representations. In either case, computational output does not acquire epistemic authority simply because it is technically sophisticated or linguistically persuasive: predictive output is not explanation, and generative output is not interpretation by default.

Emerging methodological scholarship reflects substantial disagreement regarding the appropriate role of GenAI in qualitative and interpretive research, ranging from structured approaches to AI-assisted analytical dialogue to more fundamental concerns about reflexivity, meaning-making, and epistemic authority (Jones, 2025; Friese, 2026; Greenhalgh, 2026; Nguyen and Welch, 2026). The MMCTF does not require this debate to be resolved through either unconditional adoption or categorical prohibition. Instead, it asks whether the analytical function assigned to AI is coherent with the study’s epistemological position, methodological tradition, research design, integration strategy, and intended knowledge claims.

This places particular importance on the human - AI division of analytical labour. Researchers should specify which analytical-support functions were delegated to AI, which interpretations and decisions remained human, how consequential AI-generated outputs affected subsequent analytical choices, and how those outputs were evaluated. An AI-mediated analytical audit trail can make these relationships traceable without requiring exhaustive reporting of every interaction. The guiding principle is proportional transparency: the greater the epistemic and analytical influence of AI on findings, integration, or meta-inference, the greater the methodological documentation required.

Ultimately, methodological transparency is therefore more than a reporting obligation. It is what enables readers, reviewers, and editors to reconstruct the research architecture, identify consequential points of integration, evaluate the treatment of discordance, examine the human - AI division of analytical labour where applicable, and judge whether the resulting meta-inferences are methodologically defensible.

### 11.6 Beyond performative integration

The increasing institutionalization of MMR has contributed substantially to its methodological development and visibility. At the same time, greater recognition of mixed methods approaches may create incentives to adopt MMR because it is perceived as innovative, comprehensive, fundable, publishable, or institutionally expected rather than because the research problem requires the integration of different forms of evidence. We refer to this risk as performative integration: the formal inclusion of qualitative and quantitative components, and sometimes identifiable points of integration, without a sufficiently articulated epistemic, theoretical, or inferential rationale for why these components need to be brought into relationship.

Performative integration should not be confused with poor execution of an otherwise appropriate mixed methods design. A study may follow recognizable MMR procedures, identify a design, construct a joint display, and report an integrated conclusion while the underlying rationale for mixing remains weak. Conversely, a relatively simple mixed methods design may demonstrate strong coherence when each component has a clear epistemic and analytical function and their integration addresses a research problem that neither component could adequately address alone.

The MMCTF therefore positions methodological necessity before methodological complexity. The relevant question is not whether a study contains multiple methods or sophisticated integration procedures, but whether the research problem generates a defensible need for methodological plurality and whether the resulting components form a coherent research architecture. From this perspective, choosing not to conduct MMR may sometimes represent the more methodologically coherent decision.

In this sense, the MMCTF shifts the evaluative question from “Was integration performed?” to “Was integration methodologically necessary, coherent, inferentially productive, and sufficiently transparent to be critically evaluated?” This distinction becomes particularly important as MMR designs grow more complex and as AI introduces additional analytical actors and interfaces into the research process.

The future of rigorous MMR may therefore depend less on adding methodological components than on making the relationships among them more explicit, defensible, and transparent.

## 12 Limitations and future development of the framework

The MMCTF is a conceptually developed framework rather than an empirically validated assessment instrument. Its development involved conceptual identification, differentiation, relational synthesis, and stress-testing against methodological tensions such as discordance, epistemic plurality, multiple points of integration, psychological research contexts, and AI-mediated analytical processes. This conceptual stress-testing should not be interpreted as empirical validation. Several limitations and corresponding directions for future research therefore require consideration.

First, the five MMCTF domains are analytically distinguishable but relationally interdependent. Epistemological assumptions may influence theoretical framing, research questions, design choices, analytical procedures, integration, and the scope of resulting inferences. Similarly, transparency operates across rather than independently of the other domains. The five domains should therefore not be understood as autonomous methodological compartments or as additive dimensions in which an overall coherence score can simply be calculated. Their primary purpose is to make consequential relationships within the research architecture available for critical examination.

Second, the framework risks becoming overly prescriptive if converted into a universal checklist. This would be particularly problematic because the MMCTF is intended to resist rather than reproduce performative methodological complexity. Not every MMR study requires extensive discussion of ontology, epistemology, axiology, every potential point of integration, or every possible contextual boundary condition. The appropriate level of methodological explication should be proportional to the epistemic and integrative complexity of the study and to the claims being advanced. Consequently, the absence of extensive philosophical terminology should not itself be interpreted as evidence of epistemic incoherence.

Third, coherence is partly discipline-, tradition-, and purpose-sensitive. Conventions concerning evidence, theory, explanation, causality, interpretation, and generalization vary both within psychological science and across fields such as health research, education, sociology, and implementation science. Even within qualitative inquiry, phenomenological, grounded theory, ethnographic, narrative, and other traditions assign different methodological significance to concepts such as interpretation, context, researcher positionality, and theory development. The MMCTF therefore requires further examination across methodological traditions and disciplinary contexts before claims about its broader transferability can be made.

Fourth, the distinction between coherence and convergence requires empirical and methodological elaboration. The MMCTF proposes that discordance between qualitative and quantitative findings may represent either correctable incoherence or productive methodological tension. However, distinguishing between these possibilities is not necessarily straightforward. Researchers may disagree about whether an apparent contradiction reflects differences in constructs, measurement, sampling, temporal processes, contexts, levels of analysis, or genuinely competing accounts of the phenomenon. Future applications of the framework should therefore examine whether the proposed distinction between correctable incoherence and productive discordance can support consistent and useful methodological judgements across different MMR designs.

Fifth, ecological coherence will not have identical relevance or expression across all research designs. Controlled psychological experiments can be methodologically rigorous without attempting to reproduce everyday life directly, and laboratory control may itself be necessary for particular forms of causal inference. The MMCTF therefore does not treat naturalism as a universal quality criterion. Rather, ecological coherence concerns whether the scope and boundary conditions of the meta-inference are consistent with the contextual evidence and research aims. Future work should examine how this principle operates across experimental, observational, clinical, longitudinal, experience-sampling, and other psychological research designs.

Sixth, the AI-related extension of the MMCTF necessarily addresses a rapidly developing and methodologically contested area. GenAI/LLMs and predictive AI/machine-learning approaches may perform substantially different analytical functions, and contemporary systems increasingly combine generative, predictive, classificatory, and retrieval-based capabilities. The distinction introduced in this article is therefore primarily functional rather than ontological. Future technological developments may require these categories and their methodological implications to be reconsidered.

Moreover, current methodological scholarship ranges from structured proposals for AI-assisted analytical inquiry to substantial concerns regarding reflexivity, interpretive presence, bias, provenance, and epistemic authority (Jones, 2025; Friese, 2026; Greenhalgh, 2026; Nguyen and Welch, 2026). The MMCTF consequently does not treat AI-mediated integration as an established best practice. AI-generated codes, classifications, summaries, comparisons, or explanations should instead be regarded as candidate analytical outputs whose evidential status depends on the research architecture, human engagement with the underlying evidence, and subsequent validation. The framework itself does not establish that an AI-supported procedure is valid merely because that procedure can be made coherent and transparent.

### 12.1 Future development and empirical evaluation

Future development of the MMCTF should proceed from conceptual framework toward empirical evaluation, rather than prematurely transforming the five domains into a standardized scoring instrument. An initial step could involve applying the framework retrospectively to purposively selected published MMR studies representing different psychological subfields, methodological traditions, and mixed methods designs. Such analyses could examine whether independent researchers can identify vertical and horizontal coherence, points of integration, methodological friction, contextual boundary conditions, and the relationship between integrated findings and meta-inferences.

A subsequent step could examine the framework’s usefulness prospectively during study design. Research teams could use the MMCTF to articulate the relationships among the research problem, epistemological positioning, theoretical framework, research questions, MMR purpose and design, methodological strands, points of integration, and intended meta-inferences before data collection. Comparing these prospective research architectures with the studies ultimately conducted could help determine whether the framework identifies consequential methodological tensions early enough to improve design decisions.

Further research should also examine the MMCTF as an evaluative and reflexive tool for researchers, reviewers, and editors. Of particular interest would be whether different users identify similar coherence problems and productive tensions when applying the framework to the same study. Such work could inform subsequent development of reporting guidance, decision heuristics, or appraisal questions. However, inter-rater agreement alone should not become the defining criterion for the framework’s usefulness, because some methodological disagreements may themselves reveal legitimate epistemic or disciplinary differences.

The relationship between the MMCTF and existing integration-quality and reporting frameworks also warrants systematic examination. In particular, future research could investigate how the MMCTF complements the MMIQF (Fàbregues et al., 2024), established integration strategies and joint displays, contemporary meta-inference frameworks (Younas et al., 2025), and the ongoing development of updated MMR reporting guidance. Such comparative work could determine which dimensions are already adequately addressed by existing frameworks and where the MMCTF provides additional explanatory or evaluative value. This is important to avoid unnecessary methodological proliferation.

### 12.2 AI-mediated integration as a future research agenda

A particularly important research direction concerns what may be termed AI-mediated integration validity: under what conditions, and for which methodological purposes, can AI-supported connecting, building, transformation, comparison, merging, or synthesis contribute defensible methodological value beyond - or in combination with - human-led integration?

This question should not be reduced to comparisons of whether humans and AI produce similar codes, classifications, or summaries. Such agreement may be informative but does not establish epistemic or integrative validity. Future studies should examine whether AI-supported procedures preserve theoretically important ambiguity, identify or obscure negative and discordant cases, alter the weighting of qualitative and quantitative evidence, influence the construction of joint displays, change researchers’ interpretations of methodological friction, or affect the scope and defensibility of resulting meta-inferences.

Different forms of AI also require different validation strategies. For predictive AI and machine-learning components, relevant considerations may include model performance, calibration, external validity, subgroup performance, bias, and the relationship between predictive output and substantive explanation. For GenAI/LLMs, evaluation may additionally require attention to provenance, stability across interactions, empirical grounding, preservation of contextual meaning, alternative interpretations, researcher reflexivity, and the human - AI division of analytical labour. In both cases, technical performance should not be treated as equivalent to methodological coherence.

The concept of an AI-mediated analytical audit trail also requires empirical development. Future research should investigate what level of documentation is sufficient for readers to reconstruct consequential human - AI interactions without producing unmanageable records of every prompt or computational operation. The principle of proportional transparency proposed here provides a starting hypothesis: documentation requirements should increase with the epistemic and analytical influence of AI on findings, integration, and meta-inferences. Whether this principle can be operationalized reliably and usefully remains an empirical question.

Finally, future research should examine whether AI can contribute not only to analysis but also to the evaluation of methodological coherence itself. AI systems might, for example, identify potential inconsistencies among research questions, theoretical assumptions, design choices, analytical procedures, and reported meta-inferences. Such applications could be useful as methodological prompts or stress-testing mechanisms. However, they would also create a recursive problem: an AI system used to evaluate coherence would itself become part of the methodological architecture whose role, assumptions, limitations, and influence require transparent human evaluation. The MMCTF therefore retains a fundamental principle across both current and future technological configurations: computational assistance may support methodological judgement, but epistemic accountability for the resulting knowledge claims remains with the human research team.

## 13 Conclusion

Mixed methods research has moved considerably beyond the simple combination of qualitative and quantitative methods. Contemporary MMR scholarship provides increasingly sophisticated guidance concerning methodological purpose, theoretical drive, design, timing, points and processes of integration, integration quality, joint displays, fit, and meta-inference. This conceptual analysis has argued that this methodological maturation creates a corresponding need to consider the coherence of the complete research architecture within which integration occurs. Integration remains necessary for MMR, but integration is necessary and insufficient for methodological coherence.

The proposed Mixed Methods Coherence and Transparency Framework (MMCTF) addresses this higher-order requirement through five interconnected domains: epistemic coherence; theoretical and design coherence; integrative coherence; inferential and ecological coherence; and methodological transparency. The framework is not intended to replace established approaches to MMR design, integration, integration quality, or meta-inference. Rather, it provides a relational architecture for examining whether the phenomenon under investigation, epistemological assumptions, theoretical framework, research questions, MMR purpose and design, methodological traditions, analytical procedures, points of integration, and resulting meta-inferences form an explicit and defensible whole.

This perspective also clarifies the relationship among alignment, integration, and coherence. Alignment concerns relationships between particular components of a study; integration concerns the interfaces through which qualitative and quantitative components are brought into relationship; coherence concerns whether these relationships and interfaces collectively constitute a defensible research architecture. Transparency is analytically distinct from each: it does not establish coherence but makes coherence available for critical evaluation.

Importantly, coherence should not be confused with convergence. Qualitative and quantitative evidence need not agree in order to belong within a coherent MMR study. Confirmation, complementarity, expansion, and discordance may all contribute to defensible meta-inferences when their relationships are explicitly investigated and methodologically justified. The MMCTF therefore distinguishes between correctable incoherence, where methodological friction reveals an unjustified mismatch within the research architecture, and productive discordance, where different methodological components generate divergent but defensible insights that enrich understanding of the phenomenon. The aim of coherence is not to eliminate epistemic or empirical tension, but to make such tension explicit, interpretable, and inferentially productive.

The framework also places methodological transparency in a more consequential role. Coherence concerns whether the parts of a mixed methods study belong together; transparency concerns whether readers can see why and how they belong together. Transparency is therefore not evidence of coherence, but a precondition for researchers, reviewers, and editors to evaluate it.

These principles become particularly consequential as AI enters the MMR research architecture. Generative AI/LLMs and predictive AI/machine-learning approaches should not be treated as methodologically interchangeable. Predictive systems may contribute classifications, predictions, trajectories, risk profiles, or other computational patterns, whereas GenAI may contribute candidate codes, summaries, comparisons, transformations, explanations, or integrative representations. Both can create additional human – AI - data interfaces and potentially new points of integration. Yet computational sophistication does not itself confer methodological legitimacy: predictive output is not explanation, and generative output is not interpretation by default.

Accordingly, the methodological question is not simply whether AI can perform a particular analytical task, but whether the function assigned to AI is coherent with the study’s epistemological position, theoretical framework, research questions, methodological traditions, integration strategy, and intended knowledge claims. Where AI materially influences analysis or integration, researchers should make the human - AI division of analytical labour sufficiently visible to permit evaluation of provenance, analytical influence, validation, and epistemic responsibility. The principle of proportional transparency follows: the greater the epistemic and analytical influence of AI on findings, integration, and meta-inference, the greater the requirement for methodological documentation and human evaluation. Human researchers remain accountable for the knowledge claims ultimately advanced.

Finally, the MMCTF challenges the assumption that increasing methodological complexity necessarily constitutes methodological progress. MMR should not be adopted simply because methodological plurality is perceived as innovative, comprehensive, or institutionally desirable, just as AI should not be incorporated because technological novelty is mistaken for methodological sophistication. Methodological necessity should precede methodological complexity. The appropriate sequence is therefore from the research problem and epistemic and theoretical rationale, through MMR purpose, design, and integration needs, to analytical procedures and, where justified, technology selection.

The central challenge for mixed methods research in psychological science is consequently unlikely to be whether researchers can combine more methods, integrate more datasets, or employ increasingly capable AI systems. It is whether increasing methodological and technological complexity can be organized into coherent, transparent, and critically assessable research architectures in which methodological innovation produces greater understanding rather than greater opacity. The MMCTF is proposed as a conceptual framework for supporting that task - not as a rigid checklist or final standard, but as an architecture for making the relationships on which defensible mixed methods knowledge depends more explicit, examinable, and methodologically accountable.
